# Exploring the Therapeutic Potential of *Moringa oleifera* Against Lung Cancer Through Network Modeling and Molecular Docking Analysis

**DOI:** 10.3390/ijms262010191

**Published:** 2025-10-20

**Authors:** Anuj Singh, Deepak Ohri, Olaf Wolkenhauer, Naveen Kumar Gautam, Shailendra Gupta, Krishna P. Singh

**Affiliations:** 1 Amity Institute of Biotechnology, Amity University, Lucknow 226028, India; anuj.singh14@s.amity.edu (A.S.); ohri_deepak@rediffmail.com (D.O.); 2 Department of Systems Biology & Bioinformatics, University of Rostock, 18051 Rostock, Germany; olaf.wolkenhauer@uni-rostock.de (O.W.); shailendra.gupta@uni-rostock.de (S.G.); 3 Department of Biomedical Engineering & Bioinformatics, Chhattisgarh Swami Vivekananda Technical University, Bhilai 491107, India; 4 Leibniz Institute for Food Systems Biology, Technical University of Munich, 85354 Freising, Germany; 5 Department of Urology and Renal Transplantation, Sanjay Gandhi Post Graduate Institute of Medical Sciences, Lucknow 226014, India; nkgautam@sgpgi.ac.in

**Keywords:** lung cancer, *Moringa oleifera*, network modeling, molecular docking, molecular dynamics

## Abstract

Lung cancer remains the leading cause of cancer-related mortality worldwide, with significant resistance to conventional therapies, highlighting the urgent need for novel therapeutic strategies. *Moringa oleifera* (*M. oleifera*), a medicinal plant rich in diverse bioactive compounds, has shown promising potential for anti-lung carcinoma activity. This study investigates the molecular mechanisms underlying the therapeutic effects of *M. oleifera* bioactive compounds for their anti-lung cancer activities through an integrated network modeling and molecular docking approach. By constructing comprehensive compound–target–lung cancer pathway networks, we aim to elucidate the multitarget pharmacology of *M. oleifera* compounds, providing valuable insights into their potential as therapeutic candidates. Computational pipeline was applied to identify 180 phytochemicals from *M. oleifera*, filtered using Lipinski’s Rule of Five and ADMET properties, resulting in 10 lead compounds followed by their potential biological target proteins in regulating lung cancer progression. We identified 80 targeted proteins involved in lung cancer, with EGFR being the most enriched in pathway enrichment analysis. In the molecular docking analysis, caffeic acid showed the highest binding score (−28.97 kcal/mol) with EGFR forming stable complex during molecular dynamics simulations compared to the known EGFR inhibitor ‘erlotinib’. The overall results suggest that caffeic acid, a key bioactive compound in *M. oleifera*, is an EGFR-mediated oncogenic signaling inhibitor for lung cancer therapy, warranting further experimental validation to translate these findings into clinical applications.

## 1. Introduction

**Figure 1:**
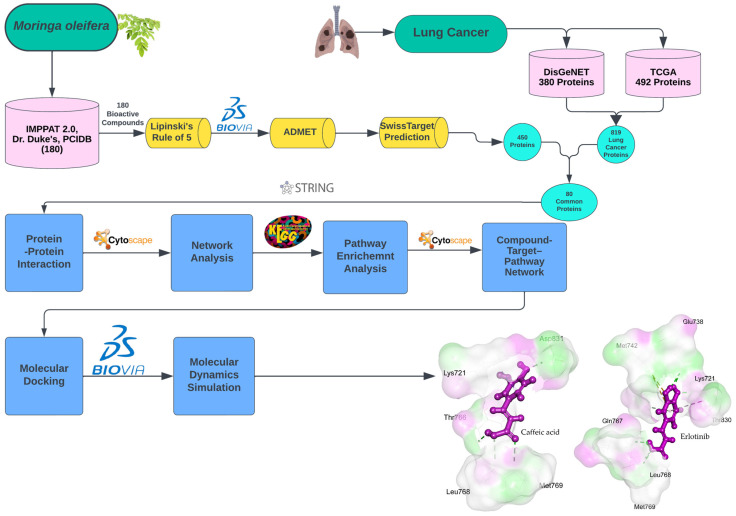
Workflow for identifying natural compounds from *M. oleifera* targeting lung cancer. Bioactive compounds were filtered using Lipinski’s Rule of Five and ADMET. Further, the potential human target proteins were identified using SwissTargetPrediction tool. We then identified lung cancer-associated proteins by combining information from DisGeNET and TCGA databases. Next the PPI network was constructed using the STRING database and analyzed using the Cytoscape software version 3.9.0 to prioritize key proteins regulating lung-cancer associated processes and pathways. Finally, we performed molecular docking and MD simulation analyses to compare binding patterns.

Lung cancer poses a substantial global public health challenge, representing the foremost cause of cancer-related fatalities worldwide, as reported by the World Health Organization (WHO). In 2022 alone, lung cancer was accountable for an estimated 1.8 million deaths, underscoring its status as the primary contributor to cancer mortality globally (source: https://gco.iarc.fr accessed on 20 December 2024) [1]. The incidence of lung cancer exhibits regional disparities, with North America, Europe, and Asia reporting the highest rates [2]. Tobacco smoking is the primary contributor to lung cancer, accounting for roughly 85% of all cases. However, other factors such as exposure to environmental pollutants, genetic susceptibility, and other risk factors also contribute. Despite the advances in treatment modalities, lung cancer prognosis remains poor, with a 5-year survival rate of less than 20% [3]. Lung cancer treatment approaches may differ based on cancer type and stage due to its complexity. Currently, lung cancer treatment options include surgery, radiation therapy, chemotherapy, targeted therapy, and immunotherapy. Chemotherapy, which employs drugs to eliminate cancer cells, is commonly administered to patients with advanced-stage lung cancer. Targeted therapy designed to pinpoint specific proteins crucial for cancer cell proliferation has emerged as a promising approach. Various targeted therapies have demonstrated encouraging outcomes in the treatment of diverse forms of lung cancer [4,5]. Despite advancements in lung cancer treatment techniques, the issue of drug resistance and the growing number of lung cancer cases in the future highlight the pressing need for the development of new medications to combat this life-threatening disease [6]. Although existing treatment modalities have made progress, their efficacy varies among patients, and the emergence of drug resistance can compromise their effectiveness. Hence, the ongoing advancement of new and potent drugs for lung cancer treatment is imperative to address the escalating incidence of this condition. Natural compounds sourced from plants are increasingly recognized as a promising avenue for discovering novel alternative anticancer drugs owing to their enhanced structural diversity, low toxicity upon metabolism in the body, and complex nature. Secondary metabolites derived from plants are particularly important primary reservoirs of medicinal agents [7,8,9,10,11,12]. *Moringa oleifera* (*M. oleifera*), commonly referred to as the drumstick tree and a staple vegetable in numerous countries since ancient times, originates from South Asia, primarily the foothills of the Himalayas, India. This tree boasts remarkable nutritional and medicinal properties [13,14,15]. *M. oleifera* has traditionally been employed to treat a myriad of ailments, including arthritis, asthma, hypertension, pain, coughs, diabetes, diarrhoea, dropsy, epilepsy, fever, headaches, hysteria, irritations, paralysis, skin infections, sores, tumors, weakness, and wounds, among others, across various countries [16,17,18]. The bioactive constituents present in almost all parts of *M. oleifera* have been associated with their efficacy against various illnesses, including cancer [19]. Recent studies have revealed that natural compounds from *M. oleifera* can exert anticancer effects [20,21]. *M. oleifera* extracts offer substantial health benefits, particularly through alcoholic and aqueous extraction of leaf compounds, which emerge as the optimal source. Their effects vary with dosage, displaying cytotoxicity towards cancer cell lines while preserving normal cell lines [22,23]. *M. oleifera* roots have demonstrated inhibitory activity against various cancer cell lines by reducing cell proliferation and inducing apoptosis [24]. Leaf extracts have been shown to significantly induce apoptosis and reduce cell proliferation in a lung cancer cell line [25]. Studies on the alkaloid extract of *M. oleifera* indicate that inhibition of JAK2/STAT3 pathway activation is the mechanism by which the extract suppresses cell migration and proliferation, induces apoptosis, and halts cell cycle progression in lung cancer [26]. This study pioneered an innovative approach employing a network modeling-based strategy to identify active natural compounds from *M. oleifera* with potential therapeutic applications in lung cancer treatment. Molecular docking and simulation techniques were used to assess the anti-lung cancer properties of *M. oleifera* active compounds, initiating the exploration of promising drug candidates derived from this plant. From a broader perspective, network modeling enables the examination of the intricate interplay between identified phytochemicals and various targets within the pathways associated with lung cancer [27,28,29]. Molecular docking was employed to elucidate the binding patterns of selected phytochemicals from *M. oleifera* with molecular targets linked to lung cancer. These analyses shed light on the mode of action of *M. oleifera* bioactive compounds in influencing cancer-related biological processes. Nevertheless, further validation of these findings through in vitro and in vivo studies is required. The detailed workflow of our study is shown in Figure 1.

## 2. Results

### 2.1. Screening of Bioactive Components of M. oleifera

The phytochemical compounds of *M. oleifera* were retrieved from the following databases: Indian Medicinal Plants, Phytochemistry and Therapeutics 2.0 (IMPPAT 2.0) [30], Dr. Duke’s Phytochemical and Ethnobotanical Databases [31], and Phyto-Chemical Interactions DB [32]. We found a total of 180 unique compounds from *M. oleifera.* All the compounds were subjected to Lipinski’s Rule of Five for drug-likeness, which includes (i) a molar refractivity value of 40–130; (ii) Log P less than 5; (iii) a number of hydrogen bond acceptors less than 10; (iv) a number of hydrogen bond donors less than 10; and (v) a molecular mass of less than 500 Da. In total, 167 natural compounds from *M. oleifera* fulfilled the cutoff criteria of Lipinski’s Rule of Five (Table S1). These compounds were further subjected to another filtering process based on ADMET-based properties.

In ADMET, we considered different properties for the filtration of compounds, such as absorption; water solubility; CYP2D6 inhibition; hepatotoxicity; and intestinal absorption. In ADMET-based filtration, water solubility is a key factor associated with the drug bioavailability. In general, the water solubility in the range of 3 and 4 are considered optimal for the drug bioavailability. The compound with solubility level below 3 are considered low to non-soluble while those with value more than 4 are highly soluble. From the 167 *M. oleifera* compounds screened, 112 were in the water solubility range of 3 and 4. Similarly, 123 compounds were found to be with good and moderate intestinal absorption level and passes through the blood stream. Out of 167 compounds, 69 with very high or high Blood Brain Barrier (BBB) permeability level were also filtered out. Plasma Protein Binding (PPB) is also one of the key pharmacokinetics parameters which determine level of free and active molecule in the blood stream for exhibiting drug action. 98 compounds possess high PPB level (TRUE) and were filtered out. Finally, 11 compounds were found to the metabolized by CYP2D6 enzyme and 51 were found to have hepatotoxic potential. Only 14 compounds out of 167 bioactive compounds identified in *M. oleifera* fulfilled all the ADMET parameters. The list of ADMET analysis of compounds is provided in Table S2.

**Figure 2:**
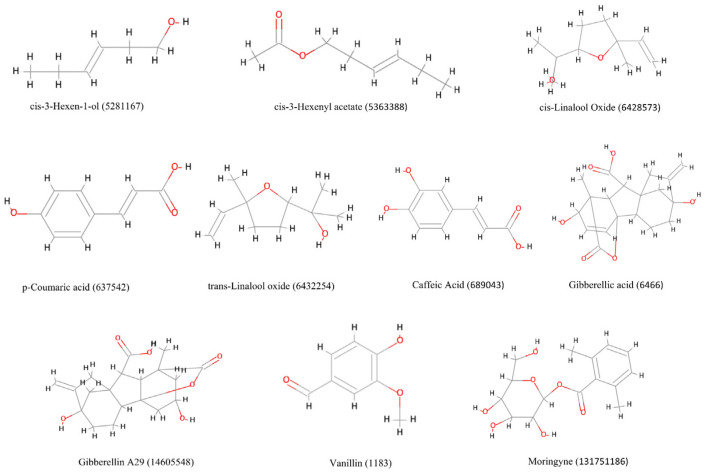
The 10 natural compounds selected after ADMET analysis using DS2022. A 2D image of these compounds, their common name, and PubChem ID are shown.

After careful screening of toxicity studies from literature and experimental data on the 14 selected compounds, we further filtered four compounds, namely ethylacetate (PubChem CID: 8857), butanol (PubChem CID: 263), benzylamine (PubChem CID: 7504), and furfural (PubChem CID: 7362) [33,34,35,36]. Therefore, in our study, we finally selected 10 compounds for further analysis, which are shown in Figure 2.

### 2.2. Potential Targets for Natural Compounds of M. oleifera and Lung Cancer

**Figure 3:**
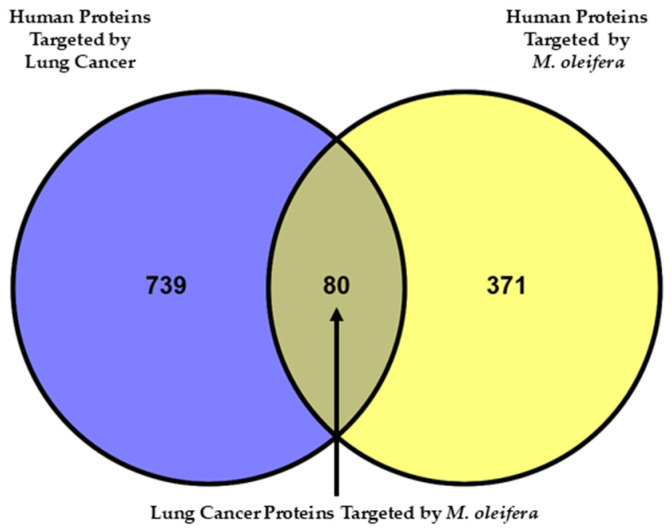
Venn diagram showing the overlap between predicted human protein targets of 10 *M. oleifera* bioactive compounds and human proteins associated with lung cancer related processes and pathways. Total of 80 lung cancer proteins are targeted by *M. oleifera* compounds.

Using the Swiss Target Prediction database, we have identified 451 unique proteins targeted for the selected 10 bioactive compounds of *M. oleifera* obtained after the ADMET analysis (Table S3). Additionally, proteins related to lung cancer were retrieved from DisGeNET (*n* = 380, Table S4) and TCGA (*n* = 492, Table S5) databases. After removing duplicates, we found 819 proteins associated with lung cancer from both databases, of which 80 proteins were targeted by *M. oleifera* compounds (Figure 3 and Table S6).

### 2.3. Protein–Protein Interaction and Network Analysis Revels Lung Cancer-Associated Hub Proteins

**Figure 4:**
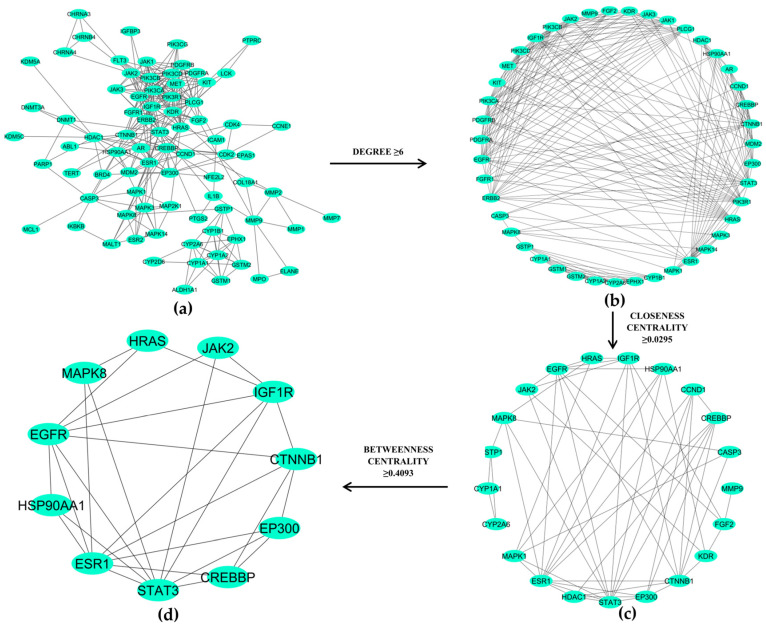
Protein–protein interaction (PPI) network of lung cancer proteins targeted by 10 *M. oleifera bioactive compounds*, consisting of (**a**) 80 nodes (proteins) and 314 edges. The network is refined using median thresholds for (**b**) degree (≥6), (**c**) closeness centrality (≥0.0295), and (**d**) betweenness centrality (≥0.4093) to identify key targets for further analysis.

A set of 80 target proteins were subjected to the STRING database with a confidence score cutoff of 0.90, and a PPI network was generated and visualized, as shown in Figure 4.

These nodes represent target proteins, and the edges indicate interactions between the proteins. The finalized PPI network was exported to Cytoscape v3.9.0 for further analysis. The Cytoscape Network Analyzer tool was used to evaluate the topological parameters of proteins within the network, including protein degree, betweenness centrality (BC), and closeness centrality (CC). The degree represents the number of direct connections with the nodes in the network. Similarly, BC quantifies how often a node lies on the shortest paths between other nodes, and CC evaluates the importance of a node based on its efficiency in interacting with other nodes. The Hub proteins in the PPI network were identified using median cutoff of degree (≥6) followed by CC (≥0.0295), and BC (≥0.4093). For the selection of the hub nodes based on the median-based cutoff criteria, we used the methodologies proposed by Zhao et al. and Wang et al. [37,38]. Using all these parameters, a total of 11 key proteins (STAT3, ESR1, EGFR, MAPK8, IGF1R, CTNNB1, CREBBP, HSP90AA1, HRAS, JAK2, and EP300) were identified as hub proteins (Figure 4d, and Table S7).

### 2.4. KEGG Pathway Enrichment Analysis of Hub Proteins

**Figure 5:**
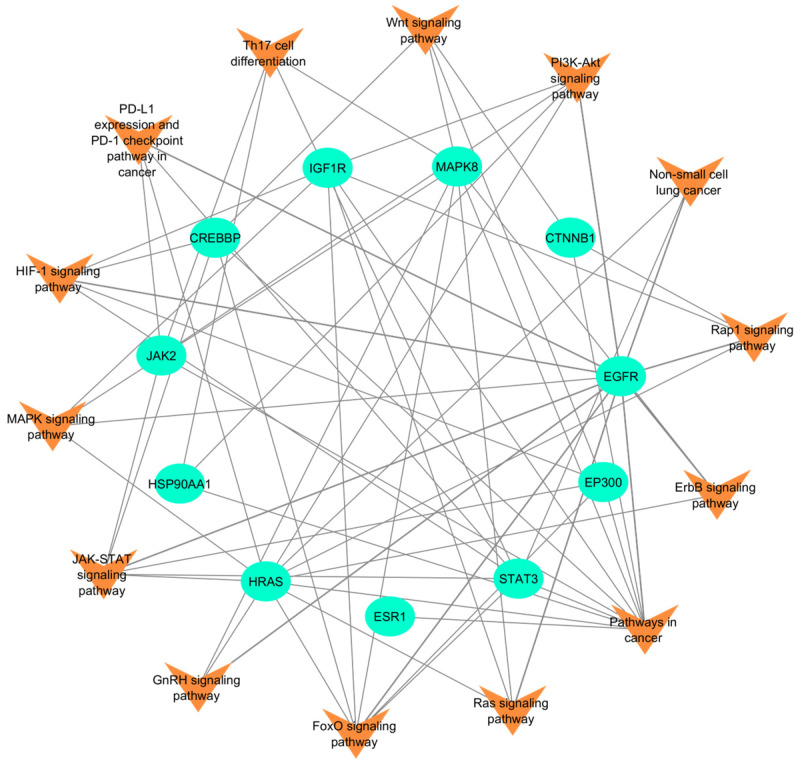
Diagram showing pathway–protein interactions from the 11 key proteins and enriched lung cancer-associated pathways. Pathways are represented in orange, whereas proteins are represented by green-colored node.

A total of 56 KEGG pathways were enriched using 11 hub proteins obtained from PPI interactions and network analysis, of which 14 pathways were associated with lung cancers (Figure 5). The JAK-STAT (Janus kinase–signal transducer and activator of transcription) signaling pathway is critical for transmitting extracellular signals to the nucleus for regulating proteins that participate in cell proliferation, differentiation, and apoptosis in lung cancer and can be used as a therapeutic lung cancer targets [39]. The FoxO (Forkhead box O) signaling pathway is involved in various cellular processes, including apoptosis, cell cycle regulation, oxidative stress resistance, and metabolism. In lung cancer, deregulation of this pathway can contribute to the development and progression of cancer [40]. The mitogen-activated protein kinase (MAPK) signaling pathway, often due to mutations in upstream receptors like EGFR or in downstream components like KRAS, leads to continuous activation of MAPK signaling. This persistent activation drives uncontrolled cell division and tumor growth and enhances metastatic potential [41]. The Ras signaling pathway stimulates angiogenesis by Ras proteins, which signal through the Ras/Raf/MAPK regulate various cellular functions. Deregulation of this pathway is a common event in cancer, as Ras is the most frequently mutated oncogene in lung cancer [42]. The GnRH (gonadotropin-releasing hormone) signaling pathway, primarily known for its role in reproductive hormone regulation, has been implicated in lung cancer development through its influence on cellular proliferation and apoptosis [43]. The PI3K/AKT signaling pathway plays a significant role in the development and progression of lung cancer. It also plays a role in the tumor environment, such as in angiogenesis and inflammatory factor recruitment [44]. The ErbB signaling pathway (erythroblastic oncogene B), often through mutations and overexpression, leads to the stimulation of downstream signaling cascades like the MAPK/ERK, PI3K/AKT and JAK/STAT pathways. In turn, they collectively contribute to uncontrolled cell growth, inhibition of apoptosis, angiogenesis, and metastasis in lung cancer [45]. The HIF-1 signaling pathway plays a crucial role in lung cancer progression by acting as a master regulator of cellular responses to hypoxia; enabling low-oxygen conditions within the tumor microenvironment; and promoting tumor growth, angiogenesis, invasion, and metastasis through the activation of various downstream genes [46]. The Rap1 signaling pathway impacts lung cancer progression by regulating cell adhesion, migration, and invasion, mainly through integrin-mediated interactions [47]. In lung cancer, PD-L1 expression helps tumors evade the immune system by activating the PD-1 checkpoint, effectively “putting the brakes” on immune responses and allowing cancer cells to grow unchecked [48]. Th17 cell differentiation pathways play a significant role in lung cancer progression by promoting tumor growth, angiogenesis, and inflammation [49]. The Wnt signaling pathway plays a significant role in lung cancer development and progression by promoting tumor cell proliferation, invasion, and resistance to therapy [50].

From the protein–pathway interactions, the most prominent protein identified is EGFR, which regulates most of the lung cancer-enriched pathways (Table S8). Inhibiting EGFR can significantly disrupt key pathways involved in lung cancer.

### 2.5. Compound–Target–Pathway Network Analysis Suggest EGFR as a Top Target of M. oleifera Compounds

**Figure 6:**
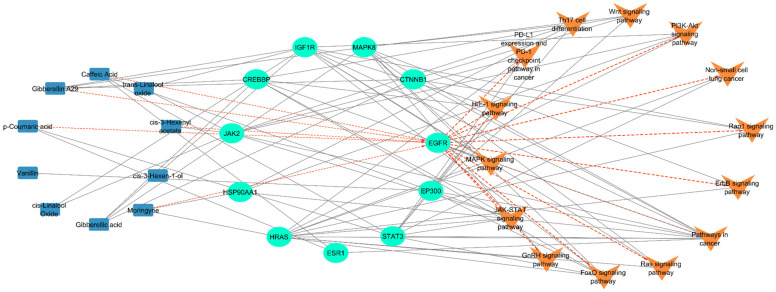
Interaction network among *M. oleifera* bioactive compounds (*n* = 10, blue nodes), target proteins (*n* = 11, green nodes), and pathways (*n* = 14, orange nodes). Interactions to and from EGFR are shown by colored arrows.

We developed a compound–target–pathway network comprising 10 *M. oleifera* bioactive compounds (Figure 6).

Our analysis revealed that EGFR is targeted by 5 out of 10 selected compounds from *M. oleifera*. The network analysis also indicates that EGFR regulates 12 out of 14 enriched pathways (Tables S8 and S9). Since most of the selected compounds regulate EGFR and most of the lung cancer pathways are regulated by EGFR, we further explored EGFR’s interaction with *M. oleifera* compounds using molecular docking analysis.

**Figure 7:**
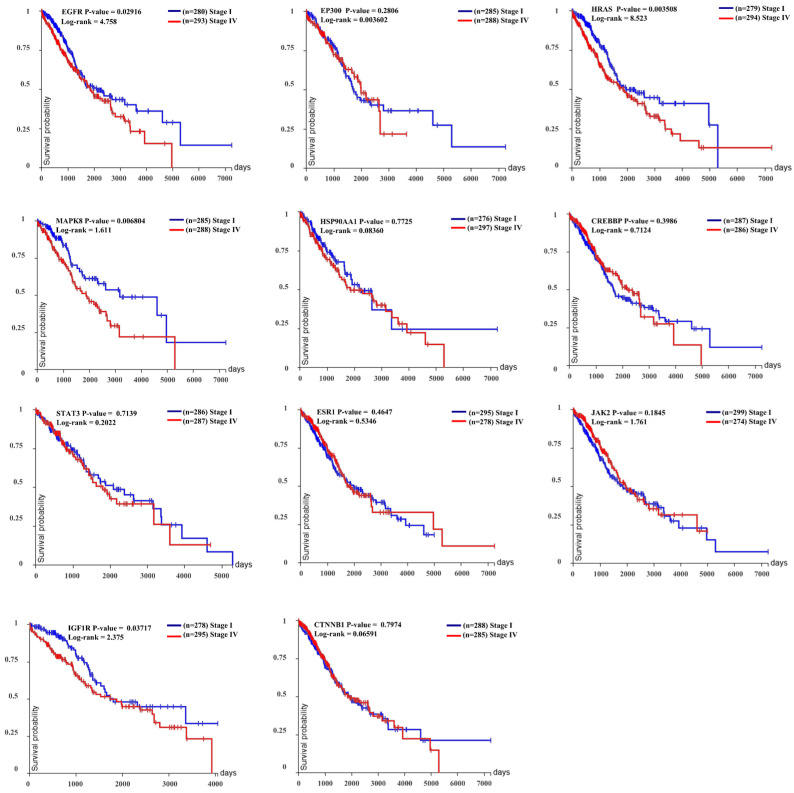
Kaplan–Meier survival curves of TCGA lung adenocarcinoma (LUAD) patients comparing Stage I (blue) and Stage IV (red) tumors for 11 key proteins (EGFR, JAK2, HRAS, MAPK8, HSP90AA1, CREBBP, STAT3, ESR1, EP300, IGF1R, and CTNNB1). The analysis stratifies survival outcomes by tumor stage to assess the prognostic relevance of each protein. n indicates the number of patients included in each stage group.

To strengthen the relevance of our computational findings and selection of biological targets, we investigated the prognostic relevance of proteins using a Kaplan–Meier survival curve in stage I versus stage IV lung adenocarcinoma (LUAD) cancer patients, analyzing 1325 NSCLC samples from TCGA data (see Figure 7).

The survival analysis was conducted using TCGA lung adenocarcinoma (LUAD) data comprising 1325 NSCLC samples across histological subtypes. Kaplan–Meier survival curves were generated to evaluate the prognostic significance of 11 key proteins (EGFR, JAK2, HRAS, MAPK8, HSP90AA1, CREBBP, STAT3, ESR1, EP300, IGF1R, and CTNNB1) by comparing Stage I and Stage IV patients. Among these, EGFR (*p* = 0.0216, Log-rank = 4.758), HRAS (*p* = 0.003808, Log-rank = 8.523), MAPK8 (*p* = 0.000594, Log-rank = 1.611), and IGF1R (*p* = 0.0371, Log-rank = 2.375) demonstrated significant survival differences, with higher expression associated with poorer outcomes and Stage I patients generally showing greater survival probability. In contrast, proteins such as EP300 (*p* = 0.2806), HSP90AA1 (*p* = 0.7725), CREBBP (*p* = 0.3986), STAT3 (*p* = 0.7139), ESR1 (*p* = 0.4617), JAK2 (*p* = 0.1848), and CTNNB1 (*p* = 0.7974) did not show statistically significant correlations with survival, which may reflect either limited biological influence on disease progression or insufficient statistical power in the current cohort. The number of patients per stratification group ranged from 274 to 386 depending on protein expression data availability, underscoring variability in sample representation. Overall, these results highlight the critical roles of EGFR, HRAS, MAPK8, and IGF1R as stage-dependent prognostic markers that may guide therapeutic decision making while also emphasizing the heterogeneity of LUAD biology within TCGA cohorts.

### 2.6. Molecular Docking Suggests Caffeic Acid a Better Inhibitors of EGFR Compared to Erlotinib

**Figure 8:**
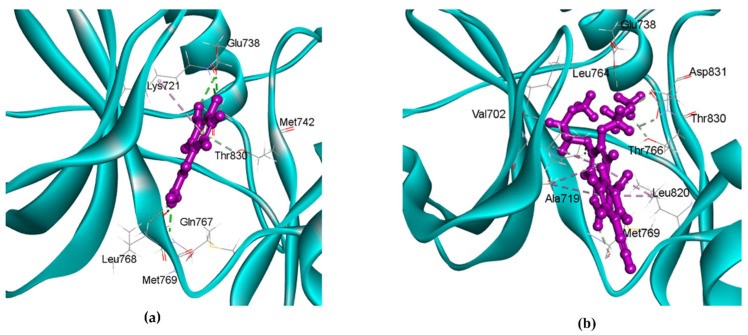
Interactions of (**a**) caffeic acid and (**b**) erlotinib in the active site of EGFR. The receptor EGFR is represented in cyan, and ligands are shown in pink. Key amino acid residues of EGFR involved in the interactions are labelled, and bonds between receptors and ligands are shown as dotted lines.

To validate our findings from the compound–protein–pathway network, we conducted molecular docking to evaluate the interactions between 10 selected bioactive compounds of *M. oleifera* and 11 lung cancer-related proteins using the CDOCKER protocol available in the Discovery Studio software suit version 2022.1.0.21297 (DS2022). CDOCKER energies of all the complexes are shown in Table S10. Interestingly, caffeic acid was identified as the best ligand for all the 11 proteins. Gibberellin A29 and Gibberellic acid did not show favorable binding with any of the target proteins. For the EGFR protein, caffeic acid exhibited the strongest binding (−28.97 kcal/mol), followed by p-coumaric acid (−26.22 kcal/mol), as detailed in Table S11 [51,52,53,54,55,56,57]. Caffeic acid formed seven hydrogen bonds and one hydrophobic interaction with active site residues of EGFR. For comparison, we performed molecular docking of experimentally validated EGFR inhibitor erlotinib. The CDOCKER energy of erlotinib was −22.64 kcal/mol, suggesting that at least two of the *M. oleifera* compounds demonstrated stronger binding scores than the experimentally validated inhibitor. To assess the robustness and reproducibility of the docking validation, we superimposed the crystallographic and redocked complexes of EGFR with erlotinib and calculated the ligand RMSD, as shown in Figure S1. The resulting RMSD was 1.381 Å, which is within the acceptable threshold (RMSD < 2.0 Å) for reliable docking accuracy [58]. The caffeic acid forms conventional hydrogen bonds with Lys721, Met769, Glu738, Gln767, Leu768, and Thr830 and undergoes hydrophobic interaction with Lys721 of EGFR, as detailed in Table S12. Notably, Met769 has previously been described as a key residue involved in EGFR inhibition, while Lys721 is known for competing with ATP and blocking kinase activity. Additionally, Glu738 helps stabilize the inactive conformation of EGFR, preventing the kinase from transitioning to its active state and thereby inhibiting its binding capability [59,60,61,62,63]. The interactions of EGFR with caffeic acid and erlotinib are shown in Figure 8, and details of all bonds are provided in Table S11. Next, we performed the 100 ns long MD simulation analysis to check the stability of docked complexes.

### 2.7. Molecular Dynamics Simulation Indicates a Stable Caffeic Acid–EGFR Complex

To evaluate the flexibility and overall stability of the docked complexes of erlotinib and caffeic acid, we performed 100 ns time-dependent MD simulations using the DS2022 software suit. More specifically, for the stability of the complexes, the root mean square deviation (RMSD) was calculated, tracking changes in atomic positions relative to their initial coordinates over time. Simultaneously, root mean square fluctuation (RMSF) was employed to assess the flexibility of individual amino acid residues by quantifying their fluctuations throughout the simulation. The structural compactness of the protein backbones was further examined using the radius of gyration (Rg), offering valuable insight into the dynamic behavior, folding, and overall compactness of the proteins under simulated biological conditions.

**Figure 9:**
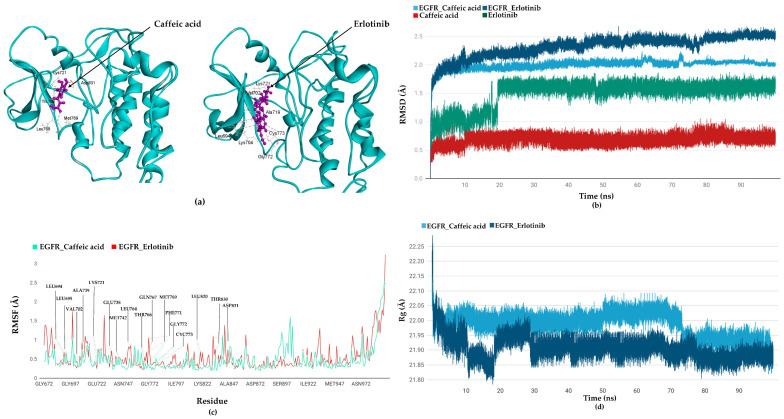
MD simulation results of the EGFR in complex with caffeic acid and erlotinib. (**a**) The final position of caffeic acid and erlotinib in the binding site of EGFR after 100 ns is shown. Both caffeic acid and erlotinib are shown in ball-and-stick mode (pink color). EGFR is shown as a solid ribbon in cyan color. Key amino acid residues involved in the binding are labelled. (**b**) Root mean square deviation (RMSD) graph of receptor and ligands highlighting deviation during the 100 ns simulation run. (**c**) Root mean square fluctuation (RMSF) of key amino acid residues within and around the EGFR active site. (**d**) Radius of gyration (Rg) of EGFR in complex with caffeic acid and erlotinib over the entire simulation run.

Figure 9a shows the final pose of the caffeic acid and erlotinib after a 100 ns MD run. In the final pose, caffeic acid had five bonds with EGFR, three of which were retained, while two were newly stabilized from the initial docking pose. Also, four bonds with Glu738, Met742, Gln767, and Thr830 initially observed in molecular docking were lost. Although the hydrogen bond with Glu738, important for stabilizing the inactive conformation of EGFR, was not consistently maintained during dynamics, compensatory interactions (e.g., with Thr766 and Asp831) together with stable RMSD (Figure 9b) and Rg (Figure 9d) values indicated that the caffeic acid–EGFR complex remained structurally stable and continued to favor the inactive state. Notably, RMSF analysis (Figure 9c) showed that Glu738 and nearby residues (Met742, Gln767, Thr830) displayed minimal fluctuations (RMSF < 1.0 Å), underscoring their local structural stability.

In the case of erlotinib, twelve bonds formed, four of which were retained, while eight were newly stabilized compared to the initial pose. Seven bonds with EGFR residues Thr766, Asp831, Glu738, Thr830, Leu764, and Met769 were lost in the final pose. All the bond information of initial and final poses of caffeic acid and erlotinib with EGFR is provided in Tables S11 and S12.

The structural deviation of the EGFR in a complex with caffeic acid and erlotinib was assessed using RMSD to evaluate the stability of the complex throughout the production run. The RMSD graph in Figure 9b shows that the EGFR in complex with caffeic acid fluctuates within a range of 1.56 to 2.00 Å only up to 10 ns, while with erlotinib, it stabilized around 40 ns in the molecular dynamics simulation. We also observed the RMSD of the ligands present in the complexes. Our results suggest that both caffeic acid and erlotinib stabilized at around 20 ns (Figure 9c). The root mean square fluctuation (RMSF) values indicate the flexibility of individual residues over a simulation run. In our study, we analyzed the fluctuations of all residues in both the complexes. Notably, the active site residues, which are critical for the stability of the docked complexes, exhibited minimal fluctuations, indicating their structural rigidity and essential role in maintaining complex stability. Specifically, in the case of erlotinib, residues Met769, Phe771, and Asp831 exhibited slightly greater fluctuations compared to those observed with caffeic acid. The low fluctuation values of these active site residues compared to the residues outside the binding cavity further indicate a favorable and stable interaction [64,65] with the caffeic acid and erlotinib. The radius of gyration (Rg) values were used to evaluate the compactness of the protein during MD simulation (Figure 9d). Our results suggest that EGFR became slightly more compact after binding with erlotinib compared to caffeic acid. Together, our results highlight that EGFR attained structural stability in the presence of both caffeic acid and erlotinib. After performing MD simulations, we calculated the interaction energies (van der Waals and electrostatic) of the ligand–EGFR complexes using the CHARMm36 forcefield to assess the binding score. The total interaction energy of the EGFR–caffeic acid complex was found to be −192.43 kcal/mol, with van der Waals and electrostatic contributions of −20.90 kcal/mol and −171.53 kcal/mol, respectively. In comparison, the EGFR–erlotinib complex exhibited a more negative total interaction energy of −244.24 kcal/mol, comprising −44.01 kcal/mol from van der Waals forces and −200.23 kcal/mol from electrostatic interactions. In contrast to the docking analyses, MD simulation results suggest that erlotinib forms slightly more stable complex with EGFR compared to caffeic acid.

**Figure 10:**
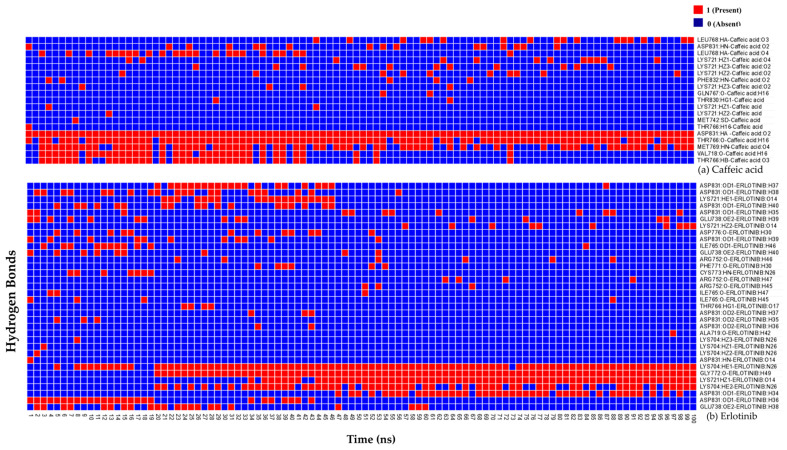
Hydrogen bond formation over the 100 ns molecular dynamics (MD) simulation. (**a**) Hydrogen bonding pattern of caffeic acid and (**b**) that of erlotinib with EGFR.

We also observed variations in the hydrogen bonds formed between EGFR amino acid residues with caffeic acid and erlotinib over the entire simulation run using DS2022 to understand the conformational changes (Figure 10).

Throughout the 100 ns MD simulation, caffeic acid maintained consistent hydrogen bond interactions with Asp831, indicating a strong and stable interaction with the active site. Previous studies by Yang et al. and Peng et al. also highlighted the role of Asp831 as a key residue involved in hydrogen bond interaction with EGFR inhibitors [66,67]. Additionally, Thr766 and Met769 also contributed significantly to caffeic acid binding through hydrogen bonds, covering approximately 90% and 66% of the simulation time. In the case of erlotinib, Lys704 and Gly772 formed hydrogen bonds for approximately 90% and 81% of the simulation time. Our results suggest that caffeic acid possesses good inhibition potential for EFGR, similar to the known inhibitor erlotinib.

## 3. Discussion

Lung cancer remains a challenging malignancy to treat, with drug resistance and adverse effects often limiting the efficacy of conventional therapies [1,68]. Nature has provided medicinal ingredients for centuries, with many modern treatments rooted in natural sources [69]. For example, morphine, derived from Papaver somniferum, is used as an analgesic [70], while taxol from *Taxus brevifolia* and vinblastine and vincristine from *Catharanthus roseus* are widely used as anticancer drugs [71,72]. Natural compounds are often favored for their lower toxicity and reduced side effects compared to synthetic drugs. For instance, natural compound Galangin, which inhibits gastric cancer, is less toxic than the commonly used chemotherapy drug Fluorouracil [73]. Similarly, Luteolin is an excellent natural active compound with minimal toxicity compared to synthetic drugs used for liver disease treatment [74]. In this study, we employed a network modeling approach to systematically investigate the therapeutic potential of *M. oleifera* against lung cancer by identifying key bioactive compounds and their associated molecular targets [75]. Our study demonstrates that *M. oleifera* bioactive compounds interact with multiple lung cancer-related proteins involved in pathways of cancer progression and metastasis. The compounds were collected from various databases and subsequently assessed for drug-likeness and ADMET properties. In drug discovery, Lipinski’s Rule of Five and ADMET analysis serve as essential tools with which to evaluate the potential oral bioavailability of molecules by examining their physicochemical characteristics as well as their absorption, distribution, metabolism, excretion, and toxicity profiles. This screening helps to identify compounds with favorable properties and increases the likelihood of successful drug candidates during early development. In this study, we elaborated upon the role of 10 *M. oleifera* compounds inhibiting lung cancer target proteins. The concentration of bioactive molecules in the plant extract has lots of therapeutic relevance. For the 10 selected bioactive compounds from *M. oleifera*, we reviewed literature for the concentration data [20,76,77,78,79,80], as shown in Table S13. We found caffeic acid as the most abundant compounds [76].

Through pathway enrichment analysis, we observed 14 pathways associated with lung cancer, with 12 linked to EGFR, 10 to HRAS, and 8 to MAPK8. Further investigation revealed that all HRAS-associated pathways were also regulated by EGFR, and six out of the eight MAPK8-associated pathways overlapped with EGFR. Given the substantial overlap, we focused our analysis on EGFR, representing the target protein with the most comprehensive set of common pathways related to lung cancer. EGFR is already known as a crucial target for lung cancer treatment, as various structural aberrations and mutations in EGFR can lead to uncontrolled tumor cell proliferation and survival [81,82]. Molecular docking was employed to investigate the interactions between EGFR and *M. oleifera* bioactive compounds identified to regulate EGFR. The docking analyses suggested that caffeic acid is the top inhibitor of EGFR among all *M. oleifera* bioactive compounds investigated in this study. Our 100 ns long MD simulation results also suggest that the binding score of the caffeic acid is almost comparable to experimentally known EGFR inhibitor erlotinib.

The comparison of ADMET properties of caffeic acid and erlotinib suggested that caffeic acid exhibited optimal solubility (level 4), a characteristic that enhances its oral bioavailability compared to erlotinib (moderate solubility). Caffeic acid possesses a low blood–brain barrier (BBB) permeability (level 3) compared to erlotinib (high BBB permeability, level 1). The BBB permeability parameter suggests that erlotinib may carry increased risk of CNS-related adverse effects, which is an issue caffeic acid may avoid due to its lower BBB permeability. Despite its absorption level being rated as 0 (good), caffeic acid showed low plasma protein binding (PPB = FALSE), suggesting a higher proportion of unbound pharmacologically active drug in circulation, thereby potentially enhancing its efficacy. Erlotinib, conversely, demonstrates high plasma protein binding (PPB = TRUE), which may reduce the effective free drug concentration. Most importantly, caffeic acid is identified as a non-hepatotoxic molecule, in contrast to erlotinib, which has been associated with hepatotoxic risks during treatment [83].

In summary, our findings highlight caffeic acid as the most promising *M. oleifera*-derived compound with strong inhibitory potential against EGFR, a key driver of lung cancer progression. Previous studies have demonstrated the anticancer properties of caffeic acid, including its ability to induce apoptosis, suppress proliferation, and modulate key signaling pathways [84,85], with some evidence also linking it to EGFR regulation [86]. Although our docking and MD simulation results indicate that erlotinib exhibits slightly stronger affinity with EGFR compared to caffeic acid, the ADMET analyses reveal important limitations of erlotinib, including moderate solubility, high blood–brain barrier permeability, hepatotoxicity, and extensive plasma protein binding, which may restrict its therapeutic window and increase the risk of adverse effects and toxicity [83,87,88]. In contrast, the overall pharmacokinetic and safety profile of caffeic acid positions it as a more favorable candidate for further development as a lung cancer therapeutic, particularly when considering the importance of balancing binding score with drug-likeness and patient safety.

## 4. Material and Methods

### 4.1. Data Mining of M. oleifera Natural Constituents

The phytochemical data of *M. oleifera* were obtained from publicly available databases such as Indian Medicinal Plants, Phytochemistry and Therapeutics 2.0 (IMPPAT 2.0) (https://cb.imsc.res.in/imppat/, accessed on 20 December 2024) [30], Dr. Duke’s Phytochemical and Ethnobotanical Databases (https://phytochem.nal.usda.gov/, accessed on 20 December 2024) [31], and Phytochemical Interactions DB (PCIDB) (https://www.genome.jp/db/pcidb/, accessed on 20 December 2024) [32]. The search outcomes were consolidated, and duplicate entries were eliminated.

### 4.2. Three-Dimensional Structure Retrieval and Molecular Property Identification

The 3D and 2D structures of the compounds were obtained from PubChem and prepared using Discovery Studio software suit version 2022.1.0.21297 (Dassault Systèmes BIOVIA, San Diego, CA, USA). The compound dataset was optimized using the smart minimizer for 1500 steps combining deepest descent followed by conjugate gradient algorithms, achieving a convergence gradient of 0.001 kcal/mol [89]. The retrieved natural compounds were screened for drug-likeness based on Lipinski’s Rule of Five [90]. This rule is a guideline in drug discovery for evaluating the potential of compounds as effective orally administered drugs. Subsequently, we assessed the absorption, distribution, metabolism, excretion, and toxicity (ADMET) properties, which are critical for drug discovery and development, using the ‘ADMET protocol’ in DS2022 [91]. In this study, specific descriptors analyzed refer specifically to ADMET (Absorption, Distribution, Metabolism, Excretion, and Toxicity) parameters, which are the physicochemical and structural properties of molecules that influence their pharmacokinetic behaviour. It included aqueous solubility, blood–brain barrier (BBB) penetration, CYP2D6 inhibition, hepatotoxicity, human intestinal absorption (HIA), and plasma protein binding (PPB). These parameters were essential for assessing the drug-likeness and therapeutic potential of the natural compounds. These standard descriptors used in pharmaceutical characterization and drug selection indicate suitability for human administration.

### 4.3. Retrieval of Targets Related to Phytochemicals and Lung Cancer

The study utilized SwissTargetPrediction to identify the potential human protein targets of the phytochemicals present in *M. oleifera* (http://www.swisstargetprediction.ch/, accessed on 25 December 2024). The SwissTargetPrediction is a web-based tool for predicting the molecular targets of bioactive small molecules in humans [92]. The selection criteria for proteins contributing to lung cancer included a two-parameter threshold: a Gene–Disease Association (GDA) score of ≥0.1 from DisGeNET v25.2 (https://www.disgenet.org; accessed on 27 December 2024) [93], and Simple Somatic Mutation (SSM)-affected cases with a mutation frequency of ≥1.06% from TCGA version 31.0 (https://portal.gdc.cancer.gov; accessed on 27 December 2024). Finally, overlapping target proteins of *M. oleifera* and lung cancer were identified for further analysis.

### 4.4. Protein–Protein Interaction (PPI) Network and Core Targets’ Identification

PPI networks are effective tools for discovering biological interactions among proteins. First, the intersected targets were considered potential therapeutic targets of the natural compounds of *M. oleifera* against lung cancer. Using the STRING Database Version 12.0 [94], the protein–protein interaction (PPI) network of shared proteins was obtained. This database provides both experimental and predicted relationship information for network contraction. We investigated the functional associations between these targets using the STRING database with a high confidence threshold (interaction score > 0.90). Network analysis visualization was conducted using Cytoscape software (version 3.9.0). In the PPI network, numerous nodes and edges represent proteins and their interactions. Nodes with a higher number of significant connections to other nodes were considered top-ranked. Three topological analyses (degree, closeness, and betweenness) were employed in the PPI network to identify core targets [95].

### 4.5. Pathway Enrichment Analysis

Gene enrichment analysis was performed using g: profiler version 0.2.3 (https://biit.cs.ut.ee/gprofiler/gost, accessed on 30 December 2024) [96] to investigate the Kyoto Encyclopedia of Genes and Genomes (KEGG) to identify lung cancer-associated pathways. From the PPI and network analysis, we identified the core proteins that were uploaded to the g: profiler tool using *Homo sapiens* as the species. We considered those pathways that played crucial roles in lung cancer and were selected from the KEGG pathways using default cutoff values (*p* < 0.05) [97].

### 4.6. Compound–Target–Pathway Network Construction

For the core protein targets identified, we prepared an integrated network by including lung cancer-specific pathways and *M. oleifera* compounds using the Cytoscape 3.9.0 [98,99]. The network was further analyzed to identify target protein and *M. oleifera* compounds that modulate most of the lung cancer-enriched pathways.

### 4.7. Molecular Docking

The top protein EGFR and *M. oleifera* compounds were subjected to molecular docking using DS2022. For this, we extracted the 3D structure of the EGFR (epidermal growth factor receptor) protein using the RCSB PDB (https://www.rcsb.org/, accessed on 20 December 2024) (PDB ID: 1M17), and the ‘Prepare Protein’ protocol of DS2022 was used to overcome the missing residues of proteins (LEU965, ALA976) which were inserted based on internal libraries and parameters described in CHARMm36 forcefield-based conformational predictions. We also addressed alternate conformations present in residues ASP831 and CYS751. For docking consistency, we retained the primary conformation (usually the ‘A’ conformation) based on occupancy. The active site of EGFR was selected around a 5 Å radius of experimentally validated ligand erlotinib present in the PDB file using the “Define and Editing” Protocol of DS2022 [59]. We generated 10 poses for each ligand predicted to target EGFR based on a pose cluster radius threshold of 0.1 Å in the binding cavity. The ligand with lowest CDOCKER energy pose was further subjected to molecular dynamics simulation together with erlotinib docking [100].

### 4.8. Molecular Dynamics Simulation

To further evaluate the binding score and stability of EGFR with the top *M. oleifera* compound caffeic acid and experimentally known inhibitor erlotinib, we performed a molecular dynamics (MD) simulation of 100 ns. More specifically, MD simulations were performed in an implicit solvent-free environment to study the stability, conformational changes, and dynamic behavior of the complexes as an inhibitor within the binding cavity of EGFR using the DS2022 protocol ‘Standard Dynamics Cascade’ [101]. The all-atom CHARMm36 forcefield [102] with dielectric constant 1 and Minimum Hydrogen Radius 0.8 was used to parameterized the protein–ligand complex to ensure that the systems simulated correctly. CHARMm36 is an academic all-atom forcefield that has previously been used for simulating proteins, nucleic acids, lipids, carbohydrates, and small molecules [103]. The steepest descent minimization algorithm was used with a maximum of 5000 steps to minimize the energy of the docked complex. Position restraints were applied to the receptor and ligand of each system for 5000 steps throughout heating (300 K) using NVT (constant temperature dynamics) and a production run of 100 ns. To analyze the structural properties of the complexes, such as root mean square deviation (RMSD), root mean square fluctuation (RMSF), and radius of gyration (Rg) between different conformations in a complete 100 ns production run, we extracted the trajectory files using ‘Process Trajectory’ followed by the ‘Analyse Trajectory’ protocol. Finally, the ‘Calculate Interaction Energy’ protocol was used to calculate the non-bonded interactions (i.e., the van der Waals and the electrostatic) between proteins and ligands for the last conformation frame of the MD simulation.

## 5. Conclusions

This research, utilizing network modeling and molecular docking methods, emphasizes the potential efficacy and molecular mechanisms of *M. oleifera* in treating lung cancer. Our findings provide preliminary evidence that compounds of *M. oleifera* interact with 80 protein targets, with compounds such as caffeic acid showing results in inhibiting EGFR and modulating critical signaling pathways associated with lung cancer compared to experimentally know EGFR inhibitor erlotinib. However, an experimental approach that encompasses both in vitro assays using EGFR-expressing lung cancer cell lines and in vivo validation—providing a comprehensive understanding of the potential therapeutic implications of caffeic acid in suppressing lung cancer metastasis—is absolutely needed.

## Data Availability

The original contributions presented in this study are included in the article/Supplementary Material, and further inquiries can be directed to the corresponding author.

## Supplementary Materials

The following supporting information can be downloaded at https://www.mdpi.com/article/10.3390/ijms262010191/s1.
