# Supplementary material for: Exploring the Therapeutic Potential of Moringa oleifera Against Lung Cancer Through Network Modeling and Molecular Docking Analysis
Source: Int J Mol Sci. 2025 Oct 20;26(20):10191. doi: 10.3390/ijms262010191 (PMC12562884; doi:10.3390/ijms262010191)
Supplement: Supplementary file 1 [file ijms-26-10191-s001.zip › ijms-3771173-supplementary.pdf]

Table S1: The table contains those 167 natural compounds from M. oleifera which filter from the Drug likeness from Lipinski's Rule of Five.

S.No.	Name	Num_H_Acceptors	Num_H_Donors	Molecular Weight	AlogP
1	3,4-dihydroxybenzoic acid	4	3	154.12	0.975
2	Benzaldehyde	1	0	106.122	1.589
3	Phenylacetaldehyde	1	0	120.149	1.516
4	Benzyl isothiocyanate	1	0	149.213	2.596
5	Trigonelline	3	0	137.136	-0.41
6	Hexanal	1	0	100.159	1.853
7	Furfural	2	0	96.0841	0.984
8	Benzylamine	1	2	107.153	0.935
9	2-Methyl-1-butanol	1	1	88.1482	1.29
10	Phenylacetonitrile	1	0	117.148	1.743
11	Ethyl Acetate	2	0	88.1051	0.369
12	Isobutyl isothiocyanate	1	0	115.197	2.204
13	Isopropyl isothiocyanate	1	0	101.17	1.739
14	3-Methyl-4,8-dihydroxy-3,4-dihydroisocoumarin	4	2	194.184	1.063
15	L-Arabinose	5	4	150.13	-2.003
16	2-Heptenal	1	0	112.17	2.286
17	2-Hexen-1-OL	1	1	100.159	1.573
18	4-Hydroxybenzoic acid	3	2	138.121	1.217
19	1-Butanol	1	1	74.1216	0.97
20	Gallic Acid	5	4	170.12	0.733
21	Octanal	1	0	128.212	2.765
22	Indole-3-acetic acid	3	2	175.184	1.788
23	Nicotinic acid	3	1	123.109	0.309
24	Oxalic Acid	4	2	90.0349	-0.429
25	Vanillin	3	1	152.147	1.33
26	D-Glucose	6	5	180.156	-2.514
27	D-Galactose	6	5	180.156	-2.514
28	Linalool	1	1	154.249	2.735
29	Camphene	0	0	136.234	2.926
30	alpha-PINENE	0	0	136.234	2.872
31	alpha-PHELLANDRENE	0	0	136.234	3.254
32	gamma-Terpinene	0	0	136.234	3.448
33	alpha-Terpinene	0	0	136.234	3.448
34	1-Methoxy-4-methylbenzene	1	0	122.164	2.299
35	1-Heptanol	1	1	116.201	2.339
36	Heptanal	1	0	114.185	2.309
37	p-Tolyl acetate	2	0	150.174	2.083
38	beta-Pinene	0	0	136.234	2.926

39	Sabinen	0	0	136.234	2.926
40	1-Octen-3-OL	1	1	128.212	2.528
41	D-Mannose	6	5	180.156	-2.514
42	Limonene	0	0	136.234	3.502
43	L-Rhamnose	5	4	164.156	-1.626
44	Myrcene	0	0	136.234	3.687
45	Perillene	1	0	150.218	3.246
46	trans-Verbenol	1	1	152.233	1.838
47	D-Glucuronic Acid	7	5	194.139	-2.386
48	Pinocarvone	1	0	150.218	1.925
49	D-Xylose	5	4	150.13	-2.003
50	3-Indoleacetonitrile	2	1	156.184	2.037
51	p-Coumaric acid	3	2	164.158	1.685
52	Anethole	1	0	148.202	2.767
53	Nerol	1	1	154.249	2.934
54	Caffeic Acid	4	3	180.157	1.443
55	cis-3-Hexen-1-ol	1	1	100.159	1.438
56	2-Hexenal	1	0	98.143	1.829
57	2-Octenal	1	0	126.196	2.742
58	2-Nonenal	1	0	140.223	3.198
59	(2R,3S,4S)-2,3,4,5-tetrahydroxypentanal	5	4	150.13	-2.28
60	(Z)-Methyl cinnamate	2	0	162.185	2.153
61	trans-Linalool oxide	2	1	170.249	1.433
62	(1R)-2-methyl-5-propan-2-ylbicyclo[3.1.0]hex-2-ene	0	0	136.234	2.872
63	Ascorbic Acid	6	4	176.124	-1.709
64	1-Octanol	1	1	130.228	2.795
65	Palmitic Acid	2	1	256.424	6.393
66	Cetyl Alcohol	1	1	242.441	6.445
67	Capric Acid	2	1	172.265	3.655
68	Lauric Acid	2	1	200.318	4.568
69	Carvone	1	0	150.218	2.501
70	Decanal	1	0	156.265	3.677
71	Dodecanal	1	0	184.318	4.59
72	1-Tetradecanol	1	1	214.387	5.532
73	4-Terpineol	1	1	154.249	2.55
74	Methyl nonanoate	2	0	172.265	3.425
75	Alpha-Terpineol	1	1	154.249	2.415
76	Nonanal	1	0	142.239	3.221
77	Dihydrozeatin	6	3	221.259	0.532
78	Epicatechin	6	5	290.268	2.021
79	Epigallocatechin	7	6	306.267	1.779
80	Rubrofusarin	5	2	272.253	2.309
81	1,3-Dibenzylurea	3	2	240.3	2.536

82	2-Cyclohexen-1-ol, 3-methyl-6-(1-methylethyl)-, (1R,6S)-rel-	1	1	154.249	2.664
83	1,7-Octadien-3-ol, 2,6-dimethyl-	1	1	154.249	2.836
84	Isopentenyladenine	5	2	203.244	1.575
85	Butin	5	3	272.253	2.373
86	Flavylium	1	0	207.247	4.248
87	ar-Turmerone	1	0	216.319	4.335
88	Ferulic acid	4	2	194.184	1.669
89	trans-Zeatin	6	3	219.243	0.485
90	Geraniol	1	1	154.249	2.934
91	trans-2,cis-6-Nonadienal	1	0	138.207	2.753
92	Neryl acetate	2	0	196.286	3.313
93	Quercetin	7	5	302.236	1.63
94	Gibberellin A1	6	3	348.39	0.617
95	Apigenin	5	3	270.237	2.41
96	Luteolin	6	4	286.236	2.168
97	Gossypetin	8	6	318.235	1.388
98	kaempferol	6	4	286.236	1.872
99	Myricetin	8	6	318.235	1.388
100	Quercetagetin	8	6	318.235	1.388
101	Ellagic Acid	8	4	302.193	1.584
102	2-Decenal	1	0	154.249	3.654
103	cis-3-Hexenyl acetate	2	0	142.196	1.817
104	(E,Z)-2,4-Decadienal	1	0	152.233	3.21
105	cis-Linalool Oxide*	2	1	170.249	1.433
106	beta-Bisabolene	0	0	204.351	5.329
107	Viridiflorol	1	1	222.366	3.202
108	(Z)-p-Menth-2-en-1-ol	1	1	154.249	2.355
109	Moringyne	7	4	312.315	0.502
110	Gibberellic acid	6	3	346.374	0.442
111	9-Octadecen-1-ol, (9Z)-	1	1	268.478	6.912
112	Cianidanol	6	5	290.268	2.021
113	Heptadecanoic acid	2	1	270.451	6.849
114	Myristic Acid	2	1	228.371	5.48
115	Tocopherols	2	1	416.68	9.954
116	Gallocatechin	7	6	306.267	1.779
117	Hesperetin	6	3	302.279	2.357
118	delta-Tocopherol	2	1	402.653	9.468
119	gamma-Tocopherol	2	1	416.68	9.954
120	Niazirin	6	3	279.288	0.46
121	Aurasperone D	10	3	556.516	4.533
122	Campesterol	1	1	400.68	7.627
123	Beta-Sitosterol	1	1	414.707	8.084
124	Arachidonic Acid	2	1	304.467	6.439

125	Palmitoleic Acid	2	1	254.408	5.948
126	Oleic Acid	2	1	282.461	6.86
127	Riboflavin	10	5	376.364	-0.255
128	6-Chromanol, 2,8-dimethyl-2-(4,8,12-trimethyltridecyl)-	2	1	402.653	9.468
129	Glucotropeolin	10	5	409.432	-0.235
130	Linoleic Acid	2	1	280.445	6.416
131	Gibberellin A20	5	2	332.391	1.719
132	Stigmasterol	1	1	412.691	7.639
133	Brassicasterol	1	1	398.664	7.183
134	5-(Heptadec-12-enyl)resorcinol	2	2	346.547	8.686
135	Oleyl alcohol	1	1	268.478	6.912
136	trans-Nerolidol	1	1	222.366	4.561
137	Stigmast-4-en-3-one	1	0	412.691	8.319
138	gamma-EUDESMOL	1	1	222.366	3.86
139	beta-Sitosterone	1	0	412.691	7.898
140	Asperglaucide	6	2	444.522	3.911
141	Niazinin	7	4	343.395	1.41
142	Niazirinin	7	2	321.325	0.839
143	Roridin E	8	1	514.607	2.795
144	Pterygospermin	4	0	406.521	7.026
145	Ergostadienol	1	1	398.664	7.87
146	5-Pentadecylresorcinol	2	2	320.509	8.219
147	24-Methylenecholesterol	1	1	398.664	7.431
148	Bauerenol	1	1	426.717	7.349
149	Campestanol	1	1	402.696	7.877
150	Stigmastanol	1	1	416.723	8.334
151	Chlorogenic Acid	9	6	354.309	-0.34
152	Clerosterol	1	1	412.691	7.888
153	Gibberellin A19	6	3	362.417	1.588
154	Zeatin riboside	10	5	351.358	-0.951
155	Niazimicin A	7	4	357.422	1.759
156	Niazimin	9	3	383.393	1.044
157	Cyanidin 3-arabinoside cation	10	7	419.359	1.619
158	Phytosterols	1	1	414.707	8.084
159	Delta 7-avenasterol	1	1	412.691	7.834
160	delta7-Avenasterol	1	1	412.691	7.834
161	cis-Zeatin riboside	10	5	351.358	-0.951
162	Gibberellin A29	6	3	348.39	0.358
163	delta-5-Avenasterol	1	1	412.691	7.834
164	niaziminin A	8	2	411.469	2.006
165	dodecahydro-1H-cyclopenta	1	1	398.664	7.431
166	28-Isoavenasterol acetate	2	0	468.754	8.419

167	2,4-Methylene cholesterol	1	1	410.675	7.757

Table S2: The table giving information about 167 natural compounds with their ADMET properties including 14 compounds that pass all the parameters are marked with symbol (*). Compounds that satisfy all the following ADMET parameter values are consider in our study: Solubility level 3 (good) or 4 (optimal); BBB Level 4 (very low), 3(Low) or 2(Medium); CYP2D6 (FALSE); Hepatotoxicity (FALSE); Absorption Level 1(moderate), 0 (good); and PPB level FALSE (Binding is>90%).

S.No.	Name	Solubility	BBB level	CYP2D6	Hepatotoxic	Adsorption level	PPB Prediction
1	3,4-dihydroxybenzoic acid	4	3	FALSE	TRUE	0	FALSE
2	benzaldehyde	4	1	FALSE	FALSE	0	TRUE
3	phenylacetaldehyde	4	1	FALSE	FALSE	0	FALSE
4	benzyl isothiocyanate	3	1	FALSE	TRUE	0	TRUE
5	Trigonelline	5	4	FALSE	TRUE	1	FALSE
6	Hexanal	4	1	FALSE	FALSE	0	FALSE
7	Furfural*	4	2	FALSE	FALSE	0	FALSE
8	Benzylamine*	4	2	FALSE	FALSE	0	FALSE
9	2-Methyl-1-butanol	4	2	FALSE	TRUE	0	FALSE
10	Phenylacetonitrile	3	1	FALSE	FALSE	0	TRUE
11	Ethyl Acetate*	4	2	FALSE	FALSE	0	FALSE
12	Isobutyl isothiocyanate	3	1	FALSE	TRUE	0	FALSE
13	Isopropyl isothiocyanate	4	1	FALSE	TRUE	0	FALSE
14	3-Methyl-4,8-dihydroxy-3,4-dihydroisocoumarin	4	3	FALSE	TRUE	0	FALSE
15	L-Arabinose	5	4	FALSE	TRUE	1	FALSE
16	2-Heptenal	3	1	FALSE	FALSE	0	TRUE
17	2-Hexen-1-OL	4	1	FALSE	FALSE	0	FALSE
18	4-Hydroxybenzoic acid	4	3	FALSE	TRUE	0	FALSE
19	1-Butanol*	4	2	FALSE	FALSE	0	FALSE
20	Gallic Acid	4	3	FALSE	TRUE	0	FALSE
21	Octanal	3	1	FALSE	FALSE	0	TRUE
22	Indole-3-acetic acid	3	2	FALSE	TRUE	0	FALSE
23	Nicotinic acid	4	3	FALSE	TRUE	0	FALSE
24	Oxalic Acid	5	3	FALSE	TRUE	0	FALSE
25	Vanillin*	4	2	FALSE	FALSE	0	FALSE
26	D-Glucose	5	4	FALSE	FALSE	3	FALSE
27	D-Galactose	5	4	FALSE	FALSE	3	FALSE
28	Linalool	3	1	FALSE	FALSE	0	TRUE
29	Camphene	3	0	FALSE	FALSE	0	TRUE
30	alpha-PINENE	3	0	FALSE	FALSE	0	TRUE
31	alpha-PHELLANDRENE	3	0	FALSE	FALSE	0	TRUE
32	gamma-Terpinene	3	0	FALSE	TRUE	0	TRUE
33	alpha-Terpinene	3	0	FALSE	FALSE	0	TRUE
34	1-Methoxy-4-methylbenzene	3	1	FALSE	FALSE	0	TRUE
35	1-Heptanol	4	1	FALSE	FALSE	0	TRUE
36	Heptanal	4	1	FALSE	FALSE	0	TRUE
37	p-Tolyl acetate	3	1	FALSE	TRUE	0	TRUE
38	beta-Pinene	3	0	FALSE	FALSE	0	TRUE
39	Sabinen	3	0	FALSE	FALSE	0	TRUE

40	1-Octen-3-OL	4	1	FALSE	FALSE	0	TRUE
41	D-Mannose	5	4	FALSE	FALSE	3	FALSE
42	Limonene	3	0	FALSE	FALSE	0	TRUE
43	L-Rhamnose	5	4	FALSE	TRUE	1	FALSE
44	Myrcene	3	0	FALSE	FALSE	0	TRUE
45	Perillene	3	1	FALSE	FALSE	0	TRUE
46	trans-Verbenol	3	1	FALSE	FALSE	0	FALSE
47	D-Glucuronic Acid	5	4	FALSE	FALSE	3	FALSE
48	Pinocarvone	3	1	FALSE	FALSE	0	TRUE
49	D-Xylose	5	4	FALSE	TRUE	1	FALSE
50	3-Indoleacetonitrile	3	2	FALSE	TRUE	0	FALSE
51	p-Coumaric acid*	4	3	FALSE	FALSE	0	FALSE
52	Anethole	3	1	FALSE	FALSE	0	TRUE
53	Nerol	3	1	FALSE	FALSE	0	TRUE
54	Caffeic Acid*	4	3	FALSE	FALSE	0	FALSE
55	cis-3-Hexen-1-ol*	4	2	FALSE	FALSE	0	FALSE
56	2-Hexenal	4	1	FALSE	FALSE	0	FALSE
57	2-Octenal	3	1	FALSE	FALSE	0	TRUE
58	2-Nonenal	3	1	FALSE	FALSE	0	TRUE
59	(2R,3S,4S)-2,3,4,5-tetrahydroxypentanal	5	4	FALSE	FALSE	3	FALSE
60	(Z)-Methyl cinnamate	3	1	FALSE	FALSE	0	TRUE
61	trans-Linalool oxide*	4	2	FALSE	FALSE	0	FALSE
62	(1R)-2-methyl-5-propan-2-ylbicyclo[3.1.0]hex-2-ene	3	0	FALSE	FALSE	0	TRUE
63	Ascorbic Acid	5	4	FALSE	FALSE	1	FALSE
64	1-Octanol	4	1	TRUE	FALSE	0	TRUE
65	Palmitic Acid	2	0	FALSE	FALSE	1	TRUE
66	Cetyl Alcohol	2	0	TRUE	FALSE	1	TRUE
67	Capric Acid	3	1	FALSE	FALSE	0	TRUE
68	Lauric Acid	3	1	FALSE	FALSE	0	TRUE
69	Carvone	3	1	FALSE	FALSE	0	TRUE
70	Decanal	3	0	FALSE	FALSE	0	TRUE
71	Dodecanal	3	0	FALSE	FALSE	0	TRUE
72	1-Tetradecanol	3	0	TRUE	FALSE	0	TRUE
73	4-Terpineol	3	1	FALSE	FALSE	0	TRUE
74	Methyl nonanoate	3	1	FALSE	FALSE	0	TRUE
75	Alpha-Terpineol	3	1	FALSE	FALSE	0	TRUE
76	Nonanal	3	1	FALSE	FALSE	0	TRUE
77	Dihydrozeatin	4	3	FALSE	TRUE	0	FALSE
78	Epicatechin	3	4	FALSE	TRUE	0	FALSE
79	Epigallocatechin	3	4	FALSE	TRUE	1	FALSE
80	Rubrofusarin	3	3	FALSE	TRUE	0	TRUE
81	1,3-Dibenzylurea	3	2	FALSE	TRUE	0	TRUE
82	2-Cyclohexen-1-ol, 3-methyl-6-(1-methylethyl)-, (1R,6S)-rel-	3	1	FALSE	FALSE	0	TRUE
83	1,7-Octadien-3-ol, 2,6-dimethyl-	3	1	FALSE	FALSE	0	TRUE

84	Isopentenyladenine	3	3	FALSE	TRUE	0	FALSE
85	Butin	3	3	FALSE	TRUE	0	FALSE
86	Flavylium	2	0	FALSE	TRUE	0	TRUE
87	ar-Turmerone	2	0	FALSE	FALSE	0	TRUE
88	Ferulic acid	4	3	FALSE	FALSE	0	TRUE
89	trans-Zeatin	4	3	FALSE	TRUE	0	FALSE
90	Geraniol	3	1	FALSE	FALSE	0	TRUE
91	643731	3	1	FALSE	FALSE	0	TRUE
92	Neryl acetate	3	1	FALSE	FALSE	0	TRUE
93	Quercetin	3	4	FALSE	TRUE	1	FALSE
94	Gibberellin A1	3	3	FALSE	FALSE	0	TRUE
95	Apigenin	3	3	FALSE	TRUE	0	TRUE
96	Luteolin	3	4	TRUE	TRUE	0	FALSE
97	Gossypetin	3	4	FALSE	TRUE	3	FALSE
98	kaempferol	3	3	FALSE	TRUE	0	FALSE
99	Myricetin	3	4	FALSE	TRUE	3	FALSE
100	Quercetagetin	3	4	FALSE	TRUE	3	FALSE
101	Ellagic Acid	3	4	FALSE	TRUE	1	TRUE
102	2-Decenal	3	0	FALSE	FALSE	0	TRUE
103	cis-3-Hexenyl acetate*	4	2	FALSE	FALSE	0	FALSE
104	(E,Z)-2,4-Decadienal	3	1	FALSE	FALSE	0	TRUE
105	cis-Linalool Oxide*	4	2	FALSE	FALSE	0	FALSE
106	beta-Bisabolene	2	0	TRUE	FALSE	1	TRUE
107	Viridiflorol	2	1	FALSE	TRUE	0	TRUE
108	(Z)-p-Menth-2-en-1-ol	3	1	FALSE	FALSE	0	FALSE
109	Moringyne*	4	4	FALSE	FALSE	0	FALSE
110	Gibberellic acid*	3	3	FALSE	FALSE	0	FALSE
111	9-Octadecen-1-ol, (9Z)-	2	0	TRUE	FALSE	2	TRUE
112	Cianidanol	3	4	FALSE	TRUE	0	FALSE
113	Heptadecanoic acid	2	4	FALSE	FALSE	2	TRUE
114	Myristic Acid	2	0	FALSE	FALSE	0	TRUE
115	Tocopherols	0	4	FALSE	FALSE	3	TRUE
116	Gallocatechin	3	4	FALSE	TRUE	1	FALSE
117	Hesperetin	3	3	TRUE	TRUE	0	FALSE
118	delta-Tocopherol	0	4	FALSE	FALSE	3	TRUE
119	gamma-Tocopherol	0	4	FALSE	FALSE	3	TRUE
120	Niazirin	4	3	FALSE	TRUE	0	FALSE
121	Aurasperone D	1	4	FALSE	TRUE	2	TRUE
122	Campesterol	1	4	FALSE	FALSE	3	TRUE
123	Beta-Sitosterol	0	4	FALSE	FALSE	3	TRUE
124	Arachidonic Acid	2	0	FALSE	FALSE	1	TRUE
125	Palmitoleic Acid	2	0	FALSE	FALSE	1	TRUE
126	Oleic Acid	2	4	FALSE	FALSE	2	TRUE
127	Riboflavin	4	4	FALSE	FALSE	3	FALSE
128	6-Chromanol, 2,8-dimethyl-2-(4,8,12-trimethyltridecyl)-	0	4	FALSE	FALSE	3	TRUE
129	Glucotropeolin	3	4	FALSE	FALSE	3	FALSE

130	Linoleic Acid	2	0	FALSE	FALSE	1	TRUE
131	Gibberellin A20	3	3	FALSE	FALSE	0	TRUE
132	Stigmasterol	1	4	FALSE	FALSE	3	TRUE
133	Brassicasterol	1	4	FALSE	FALSE	3	TRUE
134	5-(Heptadec-12-enyl)resorcinol	1	4	TRUE	FALSE	3	TRUE
135	Oleyl alcohol	2	0	TRUE	FALSE	2	TRUE
136	trans-Nerolidol	3	0	TRUE	FALSE	0	TRUE
137	Stigmast-4-en-3-one	0	4	FALSE	FALSE	3	TRUE
138	gamma-EUDESMOL	2	0	FALSE	TRUE	0	TRUE
139	beta-Sitosterone	0	4	FALSE	FALSE	3	TRUE
140	Asperglaucide	2	2	FALSE	FALSE	0	FALSE
141	Niazinin	3	3	FALSE	TRUE	0	FALSE
142	Niazirinin	4	3	FALSE	TRUE	0	FALSE
143	Roridin E	2	3	FALSE	FALSE	0	TRUE
144	Pterygospermin	1	0	FALSE	TRUE	1	TRUE
145	Ergostadienol	0	4	FALSE	FALSE	3	TRUE
146	5-Pentadecylresorcinol	2	4	TRUE	FALSE	3	TRUE
147	24-Methylenecholesterol	1	4	FALSE	FALSE	3	TRUE
148	Bauerenol	0	4	FALSE	FALSE	3	TRUE
149	Campestanol	0	4	FALSE	FALSE	3	TRUE
150	Stigmastanol	0	4	FALSE	FALSE	3	TRUE
151	Chlorogenic Acid	4	4	FALSE	FALSE	3	FALSE
152	Clerosterol	0	4	FALSE	FALSE	3	TRUE
153	Gibberellin A19	3	4	FALSE	FALSE	0	TRUE
154	Zeatin riboside	4	4	FALSE	TRUE	2	FALSE
155	Niazimicin A	3	3	FALSE	TRUE	0	FALSE
156	Niazimin	3	4	FALSE	TRUE	0	FALSE
157	Cyanidin 3-arabinoside cation	3	4	FALSE	TRUE	3	FALSE
158	Phytosterols	0	4	FALSE	FALSE	3	TRUE
159	Delta 7-avenasterol	0	4	FALSE	FALSE	3	TRUE
160	delta7-Avenasterol	0	4	FALSE	FALSE	3	TRUE
161	cis-Zeatin riboside	4	4	FALSE	TRUE	2	FALSE
162	Gibberellin A29*	3	3	FALSE	FALSE	0	FALSE
163	delta-5-Avenasterol	0	4	FALSE	FALSE	3	TRUE
164	niaziminin A	3	3	FALSE	TRUE	0	FALSE
165	dodecahydro-1H-cyclopenta	1	4	FALSE	FALSE	3	TRUE
166	28-Isoavenasterol acetate	0	4	FALSE	FALSE	3	TRUE
167	2,4-Methylene cholesterol	0	4	FALSE	FALSE	3	TRUE

Table S3: Table illustrate 450 Target proteins retrieved from the SwissTarget Prediction database from the selected 10 M. oleifera compounds

S.No.	Target Protein	*M. oleifera* bioactive compounds
1	ABCB1	Moringyne,Gibberellic acid,Gibberellin A29
2	ABCC9	cis-3-Hexen-1-ol,trans-Linalool oxide,cis-Linalool Oxide
3	ABL1	Vanillin
4	ACE	Vanillin,Caffeic Acid,Gibberellic acid,Gibberellin A29
5	ACE2	Gibberellic acid,Gibberellin A29
6	ACHE	Vanillin,cis-3-Hexen-1-ol,cis-3-Hexenyl acetate
7	ACLY	Gibberellic acid,Gibberellin A29
8	ACPP	cis-3-Hexen-1-ol,trans-Linalool oxide,cis-Linalool Oxide
9	ADA	Moringyne
10	ADAM17	Moringyne
11	ADCY1	Gibberellin A29
12	ADCY5	Vanillin
13	ADK	Moringyne
14	ADORA1	Caffeic Acid,cis-3-Hexenyl acetate,Moringyne
15	ADORA2A	Caffeic Acid,Moringyne
16	ADORA2B	Caffeic Acid,Moringyne
17	ADORA3	Moringyne
18	ADRA1D	trans-Linalool oxide,cis-3-Hexenyl acetate,cis-Linalool Oxide
19	ADRA2A	cis-3-Hexenyl acetate
20	AGTR1	Gibberellic acid,Gibberellin A29
21	AHCY	cis-3-Hexen-1-ol, trans-Linalool oxide,cis-Linalool Oxide,Moringyne
22	AHCYL1	Moringyne
23	AKR1B1	p-Coumaric acid,Caffeic Acid,cis-3-Hexen-1-ol,trans-Linalool oxide,cis-Linalool Oxide,Moringyne,Gibberellic acid,Gibberellin A29
24	AKR1B10	p-Coumaric acid,Caffeic Acid
25	AKR1C1	Moringyne
26	AKR1C2	Caffeic Acid,Moringyne
27	AKR1C3	Vanillin,Caffeic Acid,Moringyne
28	AKR1C4	p-Coumaric acid,Caffeic Acid
29	ALB	Vanillin
30	ALDH1A1	Vanillin,cis-3-Hexenyl acetate
31	ALDH2	p-Coumaric acid,cis-3-Hexenyl acetate
32	ALDH3A1	trans-Linalool oxide,cis-3-Hexenyl acetate,cis-Linalool Oxide
33	ALOX15	Caffeic Acid
34	ALOX5	p-Coumaric acid,Caffeic Acid
35	ALPG	Vanillin
36	ALPL	Vanillin,p-Coumaric acid
37	AMPD1	Gibberellin A29

38	AMPD2	Gibberellic acid
39	AMPD3	Caffeic Acid,Moringyne,Gibberellic acid,Gibberellin A29
40	AMY1A	Moringyne
41	ANPEP	Gibberellic acid,Gibberellin A29
42	AOC3	Vanillin,cis-3-Hexen-1-ol,trans-Linalool oxide,cis-Linalool Oxide
43	APP	p-Coumaric acid,Caffeic Acid,trans-Linalool oxide,cis-Linalool Oxide
44	AR	trans-Linalool oxide,cis-Linalool Oxide
45	ASAH1	cis-3-Hexen-1-ol,trans-Linalool oxide,cis-Linalool Oxide
46	ASF1A	Vanillin
47	ATP2A1	Gibberellic acid
48	BACE1	Caffeic Acid,Moringyne
49	BCAT2	Gibberellic acid,Gibberellin A29
50	BCHE	Vanillin,trans-Linalool oxide,cis-3-Hexenyl acetate,cis-Linalool Oxide
51	BDKRB1	cis-3-Hexen-1-ol,trans-Linalool oxide,cis-Linalool Oxide
52	BRD2	cis-3-Hexen-1-ol,trans-Linalool oxide,cis-Linalool Oxide
53	BRD4	cis-3-Hexen-1-ol,trans-Linalool oxide,cis-Linalool Oxide
54	BRD9	cis-3-Hexenyl acetate
55	BRPF1	cis-3-Hexenyl acetate
56	CA1	Vanillin,p-Coumaric acid,Caffeic Acid,trans-Linalool oxide,cis-Linalool Oxide
57	CA12	Vanillin,p-Coumaric acid,Caffeic Acid,cis-3-Hexen-1-ol,trans-Linalool oxide,cis-Linalool Oxide
58	CA13	cis-3-Hexen-1-ol,Benzylamine, 1-Butanol,p-Coumaric acid,Caffeic Acid,trans-Linalool oxide,cis-Linalool Oxide,Moringyne
59	CA14	Vanillin,p-Coumaric acid,Caffeic Acid,cis-3-Hexen-1-ol,trans-Linalool oxide,cis-Linalool Oxide,Moringyne
60	CA2	Vanillin,p-Coumaric acid,Caffeic Acid,cis-3-Hexen-1-ol,trans-Linalool oxide,cis-Linalool Oxide
61	CA3	Benzylamine,Ethyl Acetate,Vanillin,p-Coumaric acid,Caffeic Acid,cis-3-Hexen-1-ol,trans-Linalool oxide,cis-Linalool Oxide
62	CA4	Vanillin,p-Coumaric acid,Caffeic Acid,cis-3-Hexen-1-ol,trans-Linalool oxide,cis-Linalool Oxide
63	CA5A	Vanillin,p-Coumaric acid,Caffeic Acid,cis-3-Hexen-1-ol
64	CA5B	Vanillin,p-Coumaric acid,Caffeic Acid,cis-3-Hexen-1-ol,trans-Linalool oxide,cis-Linalool Oxide
65	CA6	Vanillin,p-Coumaric acid,Caffeic Acid,cis-3-Hexen-1-ol,trans-Linalool oxide,cis-Linalool Oxide
66	CA7	cis-3-Hexen-1-ol,Benzylamine, Vanillin,p-Coumaric acid,Caffeic Acid,trans-Linalool oxide,cis-Linalool Oxide,Moringyne
67	CA9	Vanillin,p-Coumaric acid,Caffeic Acid,trans-Linalool oxide,cis-Linalool Oxide
68	CAPN1	p-Coumaric acid
69	CAPN2	p-Coumaric acid
70	CASP1	cis-3-Hexen-1-ol,trans-Linalool oxide,cis-Linalool Oxide
71	CASP3	Gibberellic acid
72	CCNA1	Moringyne
73	CCNA2	Moringyne
74	CCNB1	cis-3-Hexen-1-ol, Moringyne
75	CCNB3	trans-Linalool oxide,cis-3-Hexenyl acetate,cis-Linalool Oxide
76	CCND1	p-Coumaric acid,Moringyne

77	CCNE1	p-Coumaric acid,
78	CDA	Moringyne
79	CDC25A	trans-Linalool oxide,cis-Linalool Oxide,Moringyne
80	CDC25B	Vanillin,Gibberellic acid
81	CDC7	cis-3-Hexen-1-ol,trans-Linalool oxide,cis-3-Hexenyl acetate,cis-Linalool Oxide
82	CDK1	cis-3-Hexen-1-ol,cis-3-Hexenyl acetate,Moringyne
83	CDK2	trans-Linalool oxide,cis-3-Hexenyl acetate,cis-Linalool Oxide,Moringyne
84	CDK5R1	cis-3-Hexen-1-ol,trans-Linalool oxide,cis-Linalool Oxide
85	CDK9	Moringyne
86	CES1	Vanillin,cis-3-Hexen-1-ol,cis-3-Hexenyl acetate
87	CES2	cis-3-Hexen-1-ol,trans-Linalool oxide,cis-3-Hexenyl acetate,cis-Linalool Oxide,Gibberellic acid,Gibberellin A29
88	CHEK1	cis-3-Hexen-1-ol
89	CHRM1	Vanillin,cis-3-Hexen-1-ol,trans-Linalool oxide,cis-3-Hexenyl acetate,cis-Linalool Oxide
90	CHRM2	cis-3-Hexen-1-ol,cis-3-Hexenyl acetate
91	CHRM3	cis-3-Hexen-1-ol,cis-3-Hexenyl acetate
92	CHRM4	cis-3-Hexen-1-ol,cis-3-Hexenyl acetate
93	CHRM5	cis-3-Hexen-1-ol,cis-3-Hexenyl acetate
94	CHRNA3	cis-3-Hexen-1-ol,cis-3-Hexenyl acetate
95	CHRNA7	Vanillin
96	CISD1	Vanillin
97	CLK1	p-Coumaric acid
98	CNR1	Vanillin,cis-3-Hexenyl acetate
99	CNR2	Vanillin
100	COMT	Vanillin,cis-3-Hexen-1-ol
101	CPA1	p-Coumaric acid,Caffeic Acid,Gibberellic acid,Gibberellin A29
102	CREBBP	Gibberellic acid,Gibberellin A29
103	CSNK2A1	cis-3-Hexen-1-ol,Moringyne,Gibberellic acid,Gibberellin A29
104	CTBP2	p-Coumaric acid,Caffeic Acid
105	CTNNB1	Gibberellic acid,Gibberellin A29
106	CTRB1	cis-3-Hexenyl acetate
107	CTRC	Gibberellic acid
108	CTSA	Gibberellic acid,Gibberellin A29
109	CTSB	cis-3-Hexenyl acetate
110	CTSG	cis-3-Hexenyl acetate
111	CTSH	cis-3-Hexenyl acetate
112	CTSK	cis-3-Hexenyl acetate
113	CTSL	Vanillin,cis-3-Hexenyl acetate
114	CTSV	Vanillin
115	CXCR1	Gibberellic acid
116	CYP11B1	cis-3-Hexen-1-ol,trans-Linalool oxide,cis-3-Hexenyl acetate,cis-Linalool Oxide
117	CYP11B2	cis-3-Hexen-1-ol,trans-Linalool oxide,cis-3-Hexenyl acetate,cis-Linalool Oxide
118	CYP19A1	trans-Linalool oxide,cis-Linalool Oxide,Gibberellic acid
119	CYP1A1	Caffeic Acid

120	CYP1A2	p-Coumaric acid,Caffeic Acid,cis-3-Hexenyl acetate
121	CYP1B1	Caffeic Acid,cis-3-Hexen-1-ol
122	CYP2A6	cis-3-Hexenyl acetate
123	CYP2C19	p-Coumaric acid,Caffeic Acid,trans-Linalool oxide,cis-Linalool Oxide
124	CYP2C9	p-Coumaric acid,Caffeic Acid
125	CYP2D6	trans-Linalool oxide,cis-Linalool Oxide
126	CYP3A4	p-Coumaric acid,Caffeic Acid,trans-Linalool oxide,cis-Linalool Oxide
127	CYP51A1	trans-Linalool oxide,cis-Linalool Oxide
128	DAO	Vanillin,p-Coumaric acid,Caffeic Acid,cis-3-Hexen-1-ol,trans-Linalool oxide,cis-Linalool Oxide,Moringyne
129	DBF4	p-Coumaric acid
130	DHFR	Gibberellic acid,Gibberellin A29
131	DHODH	cis-3-Hexen-1-ol
132	DPP4	Caffeic Acid,Moringyne
133	DRD2	Benzylamine,cis-3-Hexen-1-ol,trans-Linalool oxide,cis-Linalool Oxide
134	DRD4	cis-3-Hexen-1-ol,trans-Linalool oxide,cis-Linalool Oxide
135	DUSP1	Vanillin
136	DYRK1A	p-Coumaric acid
137	DYRK1B	p-Coumaric acid
138	ECE1	p-Coumaric acid,Caffeic Acid,Gibberellic acid,Gibberellin A29
139	EDNRA	cis-3-Hexen-1-ol,trans-Linalool oxide,cis-Linalool Oxide,Moringyne
140	EDNRB	Gibberellic acid,Gibberellin A29
141	EEF2K	p-Coumaric acid
142	EGFR	p-Coumaric acid,Caffeic Acid,cis-3-Hexen-1-ol,cis-3-Hexenyl acetate,Moringyne,Gibberellin A29
143	EGLN1	Gibberellic acid
144	EIF2AK2	p-Coumaric acid
145	EIF2AK3	trans-Linalool oxide,cis-Linalool Oxide
146	ELANE	p-Coumaric acid,Caffeic Acid,cis-3-Hexenyl acetate
147	EP300	Vanillin,Caffeic Acid
148	EPAS1	cis-3-Hexenyl acetate
149	EPHX1	cis-3-Hexenyl acetate
150	ERBB2	p-Coumaric acid,Caffeic Acid
151	ERN1	Vanillin
152	ESR1	p-Coumaric acid,Caffeic Acid
153	ESR2	p-Coumaric acid,Caffeic Acid
154	EWS-Fli1	cis-3-Hexen-1-ol
155	F10	Moringyne
156	F13A1	cis-3-Hexenyl acetate
157	F2	Caffeic Acid,cis-3-Hexenyl acetate,Moringyne,Gibberellic acid,Gibberellin A29
158	F2RL1	Gibberellic acid
159	F2RL3	cis-3-Hexen-1-ol
160	F3	p-Coumaric acid,Caffeic Acid
161	FAAH	cis-3-Hexen-1-ol,trans-Linalool oxide,cis-Linalool Oxide
162	FABP4	Gibberellic acid,Gibberellin A29

163	FBP1	Vanillin,p-Coumaric acid,Caffeic Acid,trans-Linalool oxide,cis-Linalool Oxide,Gibberellic acid,Gibberellin A29
164	FFAR1	Gibberellic acid,Gibberellin A29
165	FGF1	Moringyne
166	FGF2	Moringyne
167	FGFR1	trans-Linalool oxide,cis-Linalool Oxide
168	FKBP1A	cis-3-Hexen-1-ol,trans-Linalool oxide,cis-Linalool Oxide
169	FLT1	trans-Linalool oxide,cis-Linalool Oxide,Moringyne
170	FLT3	,trans-Linalool oxide,cis-Linalool Oxide
171	FNTA	Gibberellic acid
172	FNTB	Gibberellic acid
173	FOLH1	p-Coumaric acid,Moringyne,Gibberellic acid,Gibberellin A29
174	FTO	p-Coumaric acid,Caffeic Acid
175	FUCA1	Moringyne
176	FUT7	Vanillin
177	FYN	Vanillin,p-Coumaric acid,Caffeic Acid
178	GAA	Moringyne
179	GABRA1	cis-3-Hexenyl acetate,Gibberellic acid
180	GABRA2	cis-3-Hexenyl acetate
181	GABRA5	cis-3-Hexenyl acetate
182	GABRB2	Gibberellic acid
183	GABRB3	cis-3-Hexenyl acetate
184	GABRG2	cis-3-Hexenyl acetate,Gibberellic acid
185	GAPDH	Moringyne
186	GART	Gibberellic acid
187	GBA	Moringyne
188	GLB1	Moringyne
189	GLRA2	trans-Linalool oxide,cis-Linalool Oxide,Gibberellin A29
190	GPR17	p-Coumaric acid
191	GPR35	Moringyne
192	GRIK1	Caffeic Acid
193	GRK1	Moringyne
194	GRK2	cis-3-Hexen-1-ol
195	GRM2	cis-3-Hexenyl acetate
196	GRM4	cis-3-Hexenyl acetate
197	GRM5	Vanillin,cis-3-Hexenyl acetate
198	GSK3A	cis-3-Hexen-1-ol
199	GSK3B	p-Coumaric acid,cis-3-Hexen-1-ol,Moringyne
200	GSR	cis-3-Hexen-1-ol,trans-Linalool oxide,cis-3-Hexenyl acetate,cis-Linalool Oxide,Gibberellic acid,Gibberellin A29
201	GSTM1	Gibberellic acid
202	GSTM2	cis-3-Hexenyl acetate,Moringyne
203	GSTP1	cis-3-Hexenyl acetate,Moringyne
204	HCAR2	Vanillin,p-Coumaric acid,Caffeic Acid
205	HDAC1	cis-3-Hexen-1-ol, Moringyne

206	HDAC3	Vanillin
207	HDAC5	Vanillin
208	HDAC6	Vanillin
209	HDAC7	Vanillin
210	HDAC8	Vanillin
211	HK1	Moringyne
212	HK2	Moringyne
213	HMGCR	Vanillin,Gibberellic acid,Gibberellin A29
214	HMOX1	Vanillin,cis-3-Hexen-1-ol,trans-Linalool oxide,cis-Linalool Oxide
215	HPSE	Moringyne
216	HRAS	Moringyne
217	HSD11B1	p-Coumaric acid,Caffeic Acid,cis-3-Hexen-1-ol,Gibberellic acid,Gibberellin A29
218	HSD11B2	Gibberellin A29
219	HSD17B1	p-Coumaric acid
220	HSD17B2	p-Coumaric acid,cis-3-Hexen-1-ol,trans-Linalool oxide,cis-Linalool Oxide
221	HSP90A A1	Moringyne
222	HSPA1A	Gibberellic acid,Gibberellin A29
223	HSPA5	Moringyne
224	HSPA8	Moringyne
225	HTR2B	Benzylamine,Gibberellin A29
226	HTT	cis-3-Hexenyl acetate
227	ICAM1	cis-3-Hexenyl acetate,Gibberellic acid
228	IDE	Gibberellin A29
229	IDO1	Caffeic Acid,Gibberellic acid,Gibberellin A29
230	IGF1R	trans-Linalool oxide,cis-Linalool Oxide,Gibberellic acid,Gibberellin A29
231	IGFBP3	Gibberellic acid,Gibberellin A29
232	IKBKB	cis-3-Hexen-1-ol,Gibberellic acid
233	IL1B	Gibberellic acid
234	ILK	cis-3-Hexen-1-ol,Gibberellic acid
235	IMPDH1	Gibberellic acid,Gibberellin A29
236	IMPDH2	Vanillin,Gibberellic acid,Gibberellin A29
237	ITGA4	Gibberellin A29
238	ITGAL	Gibberellic acid,Gibberellin A29
239	ITGB1	Gibberellic acid,Gibberellin A29
240	ITGB2	Moringyne
241	JAK1	cis-3-Hexen-1-ol,trans-Linalool oxide,cis-3-Hexenyl acetate,cis-Linalool Oxide
242	JAK2	cis-3-Hexen-1-ol,trans-Linalool oxide,cis-3-Hexenyl acetate,cis-Linalool Oxide
243	JAK3	cis-3-Hexen-1-ol,trans-Linalool oxide,cis-3-Hexenyl acetate,cis-Linalool Oxide,Gibberellic acid
244	JUN	Gibberellic acid
245	KAT2B	cis-3-Hexenyl acetate
246	KCNA3	Gibberellic acid
247	KCNE1	trans-Linalool oxide,cis-Linalool Oxide
248	KCNJ11	cis-3-Hexen-1-ol,trans-Linalool oxide,cis-Linalool Oxide

249	KCNK2	cis-3-Hexenyl acetate
250	KCNMA 1	Vanillin,cis-3-Hexen-1-ol
251	KDM1A	cis-3-Hexen-1-ol
252	KDM2A	p-Coumaric acid,Caffeic Acid
253	KDM3A	p-Coumaric acid,Caffeic Acid,Gibberellic acid,Gibberellin A29
254	KDM4A	Vanillin,p-Coumaric acid,Caffeic Acid,trans-Linalool oxide,trans-Linalool oxide,cis-Linalool Oxide,Gibberellic acid,Gibberellin A29
255	KDM4B	Vanillin,trans-Linalool oxide,cis-Linalool Oxide
256	KDM4C	p-Coumaric acid,Caffeic Acid,Gibberellic acid,Gibberellin A29
257	KDM4D	Gibberellic acid,Gibberellin A29
258	KDM4E	p-Coumaric acid,Caffeic Acid
259	KDM5A	cis-3-Hexenyl acetate
260	KDM5B	Vanillin,trans-Linalool oxide,cis-3-Hexenyl acetate,cis-Linalool Oxide,Gibberellic acid,Gibberellin A29
261	KDM5C	Vanillin,trans-Linalool oxide,cis-Linalool Oxide
262	KDM6B	p-Coumaric acid,Caffeic Acid
263	KDR	cis-3-Hexen-1-ol,trans-Linalool oxide,cis-Linalool Oxide,Moringyne
264	KIF11	Benzylamine,trans-Linalool oxide,cis-Linalool Oxide,Gibberellin A29
265	KIT	trans-Linalool oxide,cis-Linalool Oxide,Moringyne
266	KLKB1	cis-3-Hexenyl acetate
267	KMO	p-Coumaric acid,Caffeic Acid,cis-3-Hexen-1-ol
268	LANCL2	Gibberellin A29
269	LAP3	p-Coumaric acid,Caffeic Acid
270	LCK	Vanillin,p-Coumaric acid,Caffeic Acid
271	LGALS3	Moringyne
272	LGALS9	Moringyne
273	LIPA	trans-Linalool oxide,cis-Linalool Oxide
274	LIPE	cis-3-Hexenyl acetate
275	LPAR3	cis-3-Hexen-1-ol
276	LRRK2	Vanillin,cis-3-Hexen-1-ol,trans-Linalool oxide,cis-3-Hexenyl acetate,cis-Linalool Oxide
277	LTA4H	Gibberellic acid,Gibberellin A29
278	LTB4R	Gibberellin A29
279	MALT1	cis-3-Hexenyl acetate
280	MANBA	Moringyne
281	MAOA	cis-3-Hexenyl acetate
282	MAOB	Vanillin,p-Coumaric acid,Caffeic Acid,trans-Linalool oxide,cis-3-Hexenyl acetate,cis-Linalool Oxide
283	MAP2K1	Moringyne
284	MAPK1	Vanillin,p-Coumaric acid,Caffeic Acid,Moringyne
285	MAPK10	Moringyne
286	MAPK14	cis-3-Hexen-1-ol,trans-Linalool oxide,cis-3-Hexenyl acetate,cis-Linalool Oxide
287	MAPK3	trans-Linalool oxide,cis-Linalool Oxide
288	MAPK8	Gibberellic acid,Gibberellin A29
289	MAPK9	Vanillin
290	MB	Vanillin

291	MCL1	Vanillin,cis-3-Hexenyl acetate,Moringyne
292	MDM2	Gibberellin A29
293	MET	p-Coumaric acid,Caffeic Acid
294	METAP1	Vanillin
295	MGLL	cis-3-Hexen-1-ol,trans-Linalool oxide,cis-3-Hexenyl acetate,cis-Linalool Oxide
296	MIF	p-Coumaric acid,Caffeic Acid,cis-3-Hexen-1-ol,trans-Linalool oxide,cis-Linalool Oxide
297	MKNK1	Vanillin
298	MME	Vanillin,p-Coumaric acid,Caffeic Acid,Moringyne,Gibberellic acid,Gibberellin A29
299	MMP1	Vanillin,p-Coumaric acid,Caffeic Acid,Moringyne,Gibberellic acid,Gibberellin A29
300	MMP12	Moringyne
301	MMP13	Moringyne
302	MMP14	Gibberellic acid,Gibberellin A29
303	MMP2	Vanillin,p-Coumaric acid,Caffeic Acid,trans-Linalool oxide,cis-Linalool Oxide,Gibberellic acid,Gibberellin A29
304	MMP3	Vanillin,Moringyne,Gibberellic acid,Gibberellin A29
305	MMP7	Moringyne
306	MMP8	Vanillin,p-Coumaric acid,Caffeic Acid,Moringyne,Gibberellic acid,Gibberellin A29
307	MMP9	Vanillin,p-Coumaric acid,Caffeic Acid,Moringyne,Gibberellin A29
308	MPO	Vanillin,cis-3-Hexen-1-ol,trans-Linalool oxide,cis-3-Hexenyl acetate,cis-Linalool Oxide
309	MTNR1A	cis-3-Hexen-1-ol
310	MTNR1B	cis-3-Hexen-1-ol
311	NAAA	cis-3-Hexenyl acetate
312	NAT1	Vanillin
313	NFE2L2	p-Coumaric acid,Caffeic Acid
314	NGFR	p-Coumaric acid,Caffeic Acid
315	NOS1	cis-3-Hexen-1-ol,trans-Linalool oxide,cis-Linalool Oxide
316	NOS2	Caffeic Acid,cis-3-Hexen-1-ol
317	NOX4	Moringyne
318	NQO2	p-Coumaric acid,Caffeic Acid
319	NR3C1	cis-3-Hexen-1-ol,trans-Linalool oxide,cis-Linalool Oxide,Gibberellic acid,Gibberellin A29
320	NR3C2	Gibberellic acid,Gibberellin A29
321	NUDT1	cis-3-Hexenyl acetate
322	OGA	Moringyne
323	OPRD1	Gibberellic acid
324	OPRK1	cis-3-Hexen-1-ol
325	OPRM1	cis-3-Hexen-1-ol,Gibberellin A29
326	P2RX3	Caffeic Acid,trans-Linalool oxide,cis-Linalool Oxide,Gibberellin A29
327	P2RX7	cis-3-Hexenyl acetate
328	PARP1	Vanillin,cis-3-Hexen-1-ol,trans-Linalool oxide,cis-3-Hexenyl acetate,cis-Linalool Oxide,Gibberellic acid,Gibberellin A29
329	PARP3	trans-Linalool oxide,cis-Linalool Oxide
330	PCSK7	Gibberellic acid
331	PDCD4	Caffeic Acid
332	PDE10A	trans-Linalool oxide,cis-3-Hexenyl acetate,cis-Linalool Oxide
333	PDE4D	Gibberellic acid

334	PDE5A	Vanillin
335	PDE7A	cis-3-Hexenyl acetate
336	PDE7B	cis-3-Hexenyl acetate
337	PDGFRA	cis-3-Hexenyl acetate
338	PDPK1	cis-3-Hexen-1-ol
339	PFKFB3	Moringyne
340	PGR	p-Coumaric acid,Caffeic Acid,cis-3-Hexen-1-ol,trans-Linalool oxide,cis-Linalool Oxide,Gibberellic acid,Gibberellin A29
341	PIK3CA	Vanillin,p-Coumaric acid,Caffeic Acid
342	PIK3CB	p-Coumaric acid,Caffeic Acid,trans-Linalool oxide,cis-Linalool Oxide
343	PIK3CD	Vanillin,trans-Linalool oxide,cis-3-Hexenyl acetate,cis-Linalool Oxide
344	PIK3CG	Vanillin
345	PIM1	Vanillin,cis-3-Hexenyl acetate,Gibberellic acid,Gibberellin A29
346	PIM2	Gibberellic acid,Gibberellin A29
347	PIM3	Vanillin
348	PKM	cis-3-Hexen-1-ol,trans-Linalool oxide,cis-Linalool Oxide
349	PLAA	Vanillin
350	PLAU	,cis-3-Hexenyl acetate
351	PNP	Moringyne,Gibberellic acid,Gibberellin A29
352	POLA1	Vanillin,Gibberellin A29
353	POLB	Vanillin,Gibberellin A29
354	PPARD	cis-3-Hexen-1-ol,trans-Linalool oxide,cis-Linalool Oxide
355	PPP1CA	Gibberellin A30
356	PPP1CC	Gibberellic acid,Gibberellin A29
357	PPP2CA	Gibberellic acid
358	PPP5C	Gibberellin A29
359	PREP	Gibberellin A29
360	PRKCA	Gibberellic acid,Gibberellin A29
361	PRKCD	Gibberellic acid
362	PRKCE	Caffeic Acid
363	PRKCQ	p-Coumaric acid,Caffeic Acid
364	PRKD1	Vanillin
365	PRKD2	Vanillin
366	PRKDC	Vanillin,trans-Linalool oxide,cis-Linalool Oxide
367	PRMT1	p-Coumaric acid
368	PRSS1	Gibberellic acid,Gibberellin A29
369	PSEN2	cis-3-Hexen-1-ol,cis-3-Hexen-1-ol,trans-Linalool oxide,cis-Linalool Oxide
370	PSMB5	trans-Linalool oxide,cis-Linalool Oxide
371	PTAFR	cis-3-Hexen-1-ol,trans-Linalool oxide,cis-Linalool Oxide
372	PTGDR	cis-3-Hexenyl acetate,Gibberellic acid
373	PTGDR2	p-Coumaric acid,Caffeic Acid,cis-3-Hexenyl acetate,cis-3-Hexenyl acetate,Gibberellic acid,Gibberellin A29
374	PTGER2	p-Coumaric acid
375	PTGER3	p-Coumaric acid
376	PTGER4	p-Coumaric acid

377	PTGES	Vanillin,Caffeic Acid,Gibberellic acid,Gibberellin A29
378	PTGS1	p-Coumaric acid,Caffeic Acid,cis-3-Hexen-1-ol,cis-3-Hexenyl acetate,Gibberellic acid,Gibberellin A29
379	PTGS2	p-Coumaric acid,Caffeic Acid,cis-3-Hexen-1-ol,trans-Linalool oxide,cis-Linalool Oxide,Gibberellic acid,Gibberellin A29
380	PTK2B	Caffeic Acid,cis-3-Hexenyl acetate
381	PTPN1	p-Coumaric acid,Caffeic Acid,Moringyne
382	PTPN2	p-Coumaric acid,Caffeic Acid,Moringyne
383	PTPN22	Vanillin
384	PTPRC	Vanillin,cis-3-Hexenyl acetate
385	PYGL	cis-3-Hexen-1-ol,Moringyne,Gibberellic acid,Gibberellin A29
386	PYGM	Moringyne,Gibberellic acid,Gibberellin A29
387	RELA	p-Coumaric acid,Caffeic Acid
388	REN	Gibberellic acid
389	RPS6KA5	cis-3-Hexen-1-ol
390	SELE	Gibberellic acid,Gibberellin A29
391	SELL	Gibberellic acid,Gibberellin A29
392	SELP	Gibberellic acid,Gibberellin A29
393	SERPINA6	Gibberellin A29
394	SERPINE1	Vanillin,Moringyne
395	SF3B3	Gibberellic acid
396	SIGMAR1	Benzylamine,cis-3-Hexen-1-ol,trans-Linalool oxide,cis-Linalool Oxide
397	SLC13A5	p-Coumaric acid,Caffeic Acid
398	SLC16A1	p-Coumaric acid
399	SLC22A12	Gibberellic acid
400	SLC28A2	Moringyne
401	SLC29A1	Moringyne
402	SLC2A1	Gibberellin A29
403	SLC5A1	trans-Linalool oxide,cis-Linalool Oxide,Moringyne,Gibberellic acid
404	SLC5A2	Moringyne
405	SLC5A7	cis-3-Hexen-1-ol
406	SLC6A2	cis-3-Hexen-1-ol,Benzylamine,p-Coumaric acid,Caffeic Acid,cis-3-Hexenyl acetate,Moringyne
407	SLC6A3	cis-3-Hexen-1-ol,trans-Linalool oxide,cis-3-Hexenyl acetate,cis-Linalool Oxide
408	SLC6A4	Gibberellin A29
409	SLC9A1	Vanillin
410	SORT1	Moringyne
411	SQLE	Moringyne
412	SRC	Vanillin,p-Coumaric acid,Gibberellic acid,Gibberellin A29
413	SRD5A2	Vanillin,p-Coumaric acid
414	SSTR1	Moringyne
415	SSTR2	Moringyne
416	SSTR3	Moringyne

417	SSTR4	Moringyne
418	SSTR5	Moringyne
419	STAT3	p-Coumaric acid,Caffeic Acid
420	SYK	p-Coumaric acid,Caffeic Acid
421	TAAR1	trans-Linalool oxide,cis-3-Hexenyl acetate,cis-Linalool Oxide
422	TBXAS1	cis-3-Hexen-1-ol,cis-3-Hexenyl acetate,Gibberellic acid,Gibberellin A29
423	TDP2	cis-3-Hexen-1-ol
424	TEK	Gibberellic acid,Gibberellin A29
425	TERT	cis-3-Hexenyl acetate
426	TGM2	Vanillin,cis-3-Hexenyl acetate
427	TK1	Moringyne
428	TLR4	p-Coumaric acid,Caffeic Acid,cis-3-Hexenyl acetate
429	TLR9	p-Coumaric acid
430	TNKS	cis-3-Hexenyl acetate
431	TNKS2	cis-3-Hexenyl acetate
432	TNNC1	Moringyne
433	TOP2A	cis-3-Hexen-1-ol,trans-Linalool oxide,cis-Linalool Oxide,Gibberellin A29
434	TPMT	Vanillin,p-Coumaric acid,Caffeic Acid
435	TRPA1	p-Coumaric acid,Caffeic Acid
436	TRPV1	cis-3-Hexen-1-ol
437	TRPV4	Gibberellin A29
438	TTR	Vanillin,Gibberellic acid,Gibberellin A29
439	TUBB1	Vanillin,Caffeic Acid
440	TYK2	cis-3-Hexen-1-ol,trans-Linalool oxide,cis-3-Hexenyl acetate,cis-Linalool Oxide
441	TYMP	Moringyne
442	TYMS	Vanillin,cis-3-Hexen-1-ol,Gibberellic acid,Gibberellin A29
443	UPP1	Moringyne
444	USP10	cis-3-Hexenyl acetate
445	USP13	cis-3-Hexenyl acetate
446	VCAM1	Vanillin
447	VCP	Moringyne
448	VEGFA	Moringyne
449	WEE1	Gibberellic acid,Gibberellin A29
450	XPO1	cis-3-Hexenyl acetate

Table S4: Table giving information of Lung Cancer genes extracted from DisGenet database with GDA score ≥ 0.1 with Disease_id C0684249.

S.No.	Protein	Protein Full Name	Score_gda
1	KRAS	KRAS proto-oncogene, GTPase	0.7
2	BRAF	B-Raf proto-oncogene, serine/threonine kinase	0.7
3	ERCC1	ERCC excision repair 1, endonuclease non-catalytic subunit	0.6
4	SLC22A18	solute carrier family 22 member 18	0.6
5	EGFR	epidermal growth factor receptor	0.5
6	PRKN	parkin RBR E3 ubiquitin protein ligase	0.5
7	PIK3CA	phosphatidylinositol-4,5-bisphosphate 3-kinase catalytic subunit alpha	0.5
8	BRCA2	BRCA2 DNA repair associated	0.5
9	TP53	tumor protein p53	0.5
10	TP63	tumor protein p63	0.5
11	ERCC6	ERCC excision repair 6, chromatin remodeling factor	0.43
12	ERBB2	erb-b2 receptor tyrosine kinase 2	0.4
13	PTEN	phosphatase and tensin homolog	0.4
14	EPHA3	EPH receptor A3	0.35
15	NTRK2	neurotrophic receptor tyrosine kinase 2	0.35
16	RICTOR	RPTOR independent companion of MTOR complex 2	0.33
17	CSNK2A3	casein kinase 2 alpha 3	0.31
18	IRF1	interferon regulatory factor 1	0.31
19	NTRK3	neurotrophic receptor tyrosine kinase 3	0.31
20	RAD21	RAD21 cohesin complex component	0.31
21	CTNNB1	catenin beta 1	0.3
22	DNMT1	DNA methyltransferase 1	0.3
23	MXRA5	matrix remodeling associated 5	0.3
24	MLH1	mutL homolog 1	0.3
25	RIT1	Ras like without CAAX 1	0.3
26	CCND2	cyclin D2	0.3
27	DNMT3A	DNA methyltransferase 3 alpha	0.28
28	CHEK2	checkpoint kinase 2	0.2
29	CHRNA3	cholinergic receptor nicotinic alpha 3 subunit	0.2
30	CHRNA5	cholinergic receptor nicotinic alpha 5 subunit	0.2
31	CHRNB4	cholinergic receptor nicotinic beta 4 subunit	0.2
32	CTNNA1	catenin alpha 1	0.2
33	CYP2A6	cytochrome P450 family 2 subfamily A member 6	0.2
34	AHR	aryl hydrocarbon receptor	0.2
35	ERCC2	ERCC excision repair 2, TFIIH core complex helicase subunit	0.2
36	CLPTM1L	CLPTM1 like	0.2
37	HYKK	hydroxylysine kinase	0.19
38	CDKN2B-AS1	CDKN2B antisense RNA 1	0.17
39	RBMS3	RNA binding motif single stranded interacting protein 3	0.15
40	RAD52	RAD52 homolog, DNA repair protein	0.15
41	WNK1	WNK lysine deficient protein kinase 1	0.15
42	DDR1	discoidin domain receptor tyrosine kinase 1	0.15

43	FGFR2	fibroblast growth factor receptor 2	0.14
44	KRT8	keratin 8	0.14
45	DNAJB4	DnaJ heat shock protein family (Hsp40) member B4	0.13
46	HLA-A	major histocompatibility complex, class I, A	0.13
47	HLA-B	major histocompatibility complex, class I, B	0.13
48	MAP3K20	mitogen-activated protein kinase kinase kinase 20	0.13
49	HLA-DQA1	major histocompatibility complex, class II, DQ alpha 1	0.12
50	KCNH1	potassium voltage-gated channel subfamily H member 1	0.12
51	MSH5	mutS homolog 5	0.12
52	SH2B3	SH2B adaptor protein 3	0.11
53	ADAR	adenosine deaminase RNA specific	0.11
54	VTI1A	vesicle transport through interaction with t-SNAREs 1A	0.11
55	DCBLD1	discoidin, CUB and LCCL domain containing 1	0.11
56	ZNRD1	zinc ribbon domain containing 1	0.11
57	ROCK1	Rho associated coiled-coil containing protein kinase 1	0.11
58	ZSCAN31	zinc finger and SCAN domain containing 31	0.11
59	MTMR3	myotubularin related protein 3	0.11
60	NAT2	N-acetyltransferase 2	0.1
61	HOTAIR	HOX transcript antisense RNA	0.1
62	ZSCAN16-AS1	ZSCAN16 antisense RNA 1	0.1
63	LINC00240	long intergenic non-protein coding RNA 240	0.1
64	BCL2L11	BCL2 like 11	0.1
65	LMCD1-AS1	LMCD1 antisense RNA 1	0.1
66	SLC17A4	solute carrier family 17 member 4	0.1
67	RTCA-AS1	RTCA antisense RNA 1	0.1
68	MSH5-SAPCD1	MSH5-SAPCD1 readthrough (NMD candidate)	0.1
69	FOXP4-AS1	FOXP4 antisense RNA 1	0.1
70	FRY	FRY microtubule binding protein	0.1
71	CDK2	cyclin dependent kinase 2	0.1
72	RBM5	RNA binding motif protein 5	0.1
73	CDK4	cyclin dependent kinase 4	0.1
74	MPZL2	myelin protein zero like 2	0.1
75	PLIN3	perilipin 3	0.1
76	CDKN1A	cyclin dependent kinase inhibitor 1A	0.1
77	CDKN1B	cyclin dependent kinase inhibitor 1B	0.1
78	LINC02831	long intergenic non-protein coding RNA 2831	0.1
79	LINC01828	long intergenic non-protein coding RNA 1828	0.1
80	CDKN2A	cyclin dependent kinase inhibitor 2A	0.1
81	YAP1	Yes associated protein 1	0.1
82	CEACAM5	CEA cell adhesion molecule 5	0.1
83	SCGN	secretagogin, EF-hand calcium binding protein	0.1
84	AHSA1	activator of HSP90 ATPase activity 1	0.1
85	SEPTIN14P21	septin 14 pseudogene 21	0.1

86	MTCO2P12	MT-CO2 pseudogene 12	0.1
87	CEACAM3	CEA cell adhesion molecule 3	0.1
88	PPP1R13L	protein phosphatase 1 regulatory subunit 13 like	0.1
89	CD3EAP	CD3e molecule associated protein	0.1
90	HCP5	HLA complex P5	0.1
91	CEACAM7	CEA cell adhesion molecule 7	0.1
92	GADD45G	growth arrest and DNA damage inducible gamma	0.1
93	MORF4L1	mortality factor 4 like 1	0.1
94	IL24	interleukin 24	0.1
95	KATNA1	katanin catalytic subunit A1	0.1
96	BTN3A1	butyrophilin subfamily 3 member A1	0.1
97	BTN2A1	butyrophilin subfamily 2 member A1	0.1
98	ADAMTS7	ADAM metallopeptidase with thrombospondin type 1 motif 7	0.1
99	RASSF1	Ras association domain family member 1	0.1
100	WIF1	WNT inhibitory factor 1	0.1
101	TP53TG1	TP53 target 1	0.1
102	TUSC2	tumor suppressor 2, mitochondrial calcium regulator	0.1
103	CHRNA4	cholinergic receptor nicotinic alpha 4 subunit	0.1
104	UCN3	urocortin 3	0.1
105	H3P10	H3 histone pseudogene 10	0.1
106	SDR9C7	short chain dehydrogenase/reductase family 9C member 7	0.1
107	SLCO6A1	solute carrier organic anion transporter family member 6A1	0.1
108	CRK	CRK proto-oncogene, adaptor protein	0.1
109	CRP	C-reactive protein	0.1
110	PARP1	poly(ADP-ribose) polymerase 1	0.1
111	CRYZ	crystallin zeta	0.1
112	MAPK14	mitogen-activated protein kinase 14	0.1
113	CSF2	colony stimulating factor 2	0.1
114	CSF3	colony stimulating factor 3	0.1
115	CSK	C-terminal Src kinase	0.1
116	CTLA4	cytotoxic T-lymphocyte associated protein 4	0.1
117	CTNND1	catenin delta 1	0.1
118	NEK10	NIMA related kinase 10	0.1
119	CYP1A1	cytochrome P450 family 1 subfamily A member 1	0.1
120	CYP1A2	cytochrome P450 family 1 subfamily A member 2	0.1
121	CYP1B1	cytochrome P450 family 1 subfamily B member 1	0.1
122	CYP2B6	cytochrome P450 family 2 subfamily B member 6	0.1
123	CYP2D6	cytochrome P450 family 2 subfamily D member 6	0.1
124	CYP2E1	cytochrome P450 family 2 subfamily E member 1	0.1
125	CYP3A5	cytochrome P450 family 3 subfamily A member 5	0.1
126	CYP21A2	cytochrome P450 family 21 subfamily A member 2	0.1
127	CYP24A1	cytochrome P450 family 24 subfamily A member 1	0.1
128	ACE	angiotensin I converting enzyme	0.1
129	TIMM8A	translocase of inner mitochondrial membrane 8A	0.1
130	PPP1R18	protein phosphatase 1 regulatory subunit 18	0.1
131	NQO1	NAD(P)H quinone dehydrogenase 1	0.1

132	AGER	advanced glycosylation end-product specific receptor	0.1
133	DNMT3B	DNA methyltransferase 3 beta	0.1
134	E2F1	E2F transcription factor 1	0.1
135	EGF	epidermal growth factor	0.1
136	EIF4E	eukaryotic translation initiation factor 4E	0.1
137	ELANE	elastase, neutrophil expressed	0.1
138	ENO2	enolase 2	0.1
139	EPHB2	EPH receptor B2	0.1
140	EPHX1	epoxide hydrolase 1	0.1
141	ERBB3	erb-b2 receptor tyrosine kinase 3	0.1
142	AKT1	AKT serine/threonine kinase 1	0.1
143	ERCC5	ERCC excision repair 5, endonuclease	0.1
144	ESR1	estrogen receptor 1	0.1
145	ESR2	estrogen receptor 2	0.1
146	ALB	albumin	0.1
147	EZH2	enhancer of zeste 2 polycomb repressive complex 2 subunit	0.1
148	ALDH1A1	aldehyde dehydrogenase 1 family member A1	0.1
149	FGF2	fibroblast growth factor 2	0.1
150	FGFR1	fibroblast growth factor receptor 1	0.1
151	FHIT	fragile histidine triad diadenosine triphosphatase	0.1
152	TDGF1P6	teratocarcinoma-derived growth factor 1 pseudogene 6	0.1
153	RALY	RALY heterogeneous nuclear ribonucleoprotein	0.1
154	DKK1	dickkopf WNT signaling pathway inhibitor 1	0.1
155	PDS5B	PDS5 cohesin associated factor B	0.1
156	FOXM1	forkhead box M1	0.1
157	FOXO3	forkhead box O3	0.1
158	EPB41L3	erythrocyte membrane protein band 4.1 like 3	0.1
159	TBC1D9	TBC1 domain family member 9	0.1
160	FN1	fibronectin 1	0.1
161	SIRT1	sirtuin 1	0.1
162	SMUG1	single-strand-selective monofunctional uracil-DNA glycosylase 1	0.1
163	ALK	ALK receptor tyrosine kinase	0.1
164	MTOR	mechanistic target of rapamycin kinase	0.1
165	FUS	FUS RNA binding protein	0.1
166	GABPA	GA binding protein transcription factor subunit alpha	0.1
167	TCERG1L	transcription elongation regulator 1 like	0.1
168	GALK2	galactokinase 2	0.1
169	RNF19A	ring finger protein 19A, RBR E3 ubiquitin protein ligase	0.1
170	TENM4	teneurin transmembrane protein 4	0.1
171	COPD	Pulmonary disease, chronic obstructive, severe early-onset	0.1
172	POLDIP2	DNA polymerase delta interacting protein 2	0.1
173	AK5	adenylate kinase 5	0.1
174	GBAP1	glucosylceramidase beta pseudogene 1	0.1
175	HPGDS	hematopoietic prostaglandin D synthase	0.1
176	EML4	EMAP like 4	0.1
177	NEAT1	nuclear paraspeckle assembly transcript 1	0.1

178	SETD2	SET domain containing 2, histone lysine methyltransferase	0.1
179	BABAM1	BRISC and BRCA1 A complex member 1	0.1
180	CD274	CD274 molecule	0.1
181	GSTM1	glutathione S-transferase mu 1	0.1
182	GSTM2	glutathione S-transferase mu 2	0.1
183	GSTP1	glutathione S-transferase pi 1	0.1
184	GSTT1	glutathione S-transferase theta 1	0.1
185	GULOP	gulonolactone (L-) oxidase, pseudogene	0.1
186	HDAC1	histone deacetylase 1	0.1
187	HGF	hepatocyte growth factor	0.1
188	HIF1A	hypoxia inducible factor 1 subunit alpha	0.1
189	HLA-DOB	major histocompatibility complex, class II, DO beta	0.1
190	HLA-DQA2	major histocompatibility complex, class II, DQ alpha 2	0.1
191	HLA-DRB9	major histocompatibility complex, class II, DR beta 9 (pseudogene)	0.1
192	HMGB1	high mobility group box 1	0.1
193	HNRNPA2 B1	heterogeneous nuclear ribonucleoprotein A2/B1	0.1
194	APC	APC regulator of WNT signaling pathway	0.1
195	HRAS	HRas proto-oncogene, GTPase	0.1
196	APEX1	apurinic/apyrimidinic endodeoxyribonuclease 1	0.1
197	HSPA4	heat shock protein family A (Hsp70) member 4	0.1
198	HSPB1	heat shock protein family B (small) member 1	0.1
199	HSPB2	heat shock protein family B (small) member 2	0.1
200	HSP90AA1	heat shock protein 90 alpha family class A member 1	0.1
201	ICAM1	intercellular adhesion molecule 1	0.1
202	MALRD1	MAM and LDL receptor class A domain containing 1	0.1
203	IFNG	interferon gamma	0.1
204	IGF1	insulin like growth factor 1	0.1
205	IGF1R	insulin like growth factor 1 receptor	0.1
206	IGF2	insulin like growth factor 2	0.1
207	IGFBP3	insulin like growth factor binding protein 3	0.1
208	FAS	Fas cell surface death receptor	0.1
209	IL1B	interleukin 1 beta	0.1
210	IL6	interleukin 6	0.1
211	CXCL8	C-X-C motif chemokine ligand 8	0.1
212	IL10	interleukin 10	0.1
213	IL17A	interleukin 17A	0.1
214	JAK2	Janus kinase 2	0.1
215	GSTK1	glutathione S-transferase kappa 1	0.1
216	MALAT1	metastasis associated lung adenocarcinoma transcript 1	0.1
217	LINC00533	long intergenic non-protein coding RNA 533	0.1
218	KRT19	keratin 19	0.1
219	LINC00511	long intergenic non-protein coding RNA 511	0.1
220	MIR125A	microRNA 125a	0.1
221	MIR145	microRNA 145	0.1
222	MIR146A	microRNA 146a	0.1
223	MIR155	microRNA 155	0.1

224	MIR182	microRNA 182	0.1
225	MIR205	microRNA 205	0.1
226	MIR21	microRNA 21	0.1
227	MIR210	microRNA 210	0.1
228	MIR31	microRNA 31	0.1
229	MIR34A	microRNA 34a	0.1
230	EPCAM	epithelial cell adhesion molecule	0.1
231	MCL1	MCL1 apoptosis regulator, BCL2 family member	0.1
232	MDM2	MDM2 proto-oncogene	0.1
233	MDM4	MDM4 regulator of p53	0.1
234	ME1	malic enzyme 1	0.1
235	MET	MET proto-oncogene, receptor tyrosine kinase	0.1
236	MGMT	O-6-methylguanine-DNA methyltransferase	0.1
237	MKLN1	muskelin 1	0.1
238	ASCL1	achaete-scute family bHLH transcription factor 1	0.1
239	MMP1	matrix metallopeptidase 1	0.1
240	MMP2	matrix metallopeptidase 2	0.1
241	MMP7	matrix metallopeptidase 7	0.1
242	MMP9	matrix metallopeptidase 9	0.1
243	MMP13	matrix metallopeptidase 13	0.1
244	MPO	myeloperoxidase	0.1
245	ABCC1	ATP binding cassette subfamily C member 1	0.1
246	STX17-AS1	STX17 antisense RNA 1	0.1
247	MSH3	mutS homolog 3	0.1
248	COX2	cytochrome c oxidase subunit II	0.1
249	MTHFR	methylenetetrahydrofolate reductase	0.1
250	MTX1	metaxin 1	0.1
251	MUC1	mucin 1, cell surface associated	0.1
252	MYC	MYC proto-oncogene, bHLH transcription factor	0.1
253	ATM	ATM serine/threonine kinase	0.1
254	NFE2L2	nuclear factor, erythroid 2 like 2	0.1
255	NME1	NME/NM23 nucleoside diphosphate kinase 1	0.1
256	NOTCH1	notch receptor 1	0.1
257	OGG1	8-oxoguanine DNA glycosylase	0.1
258	SERPINE1	serpin family E member 1	0.1
259	FOXP3	forkhead box P3	0.1
260	PCNA	proliferating cell nuclear antigen	0.1
261	PDCD1	programmed cell death 1	0.1
262	WWOX	WW domain containing oxidoreductase	0.1
263	ABCB1	ATP binding cassette subfamily B member 1	0.1
264	SERPINA1	serpin family A member 1	0.1
265	PIK3CB	phosphatidylinositol-4,5-bisphosphate 3-kinase catalytic subunit beta	0.1
266	PIK3CD	phosphatidylinositol-4,5-bisphosphate 3-kinase catalytic subunit delta	0.1
267	PIK3CG	phosphatidylinositol-4,5-bisphosphate 3-kinase catalytic subunit gamma	0.1
268	ETAA1	ETAA1 activator of ATR kinase	0.1
269	POU5F1	POU class 5 homeobox 1	0.1

270	CROT	carnitine O-octanoyltransferase	0.1
271	PPARG	peroxisome proliferator activated receptor gamma	0.1
272	GIPC2	GIPC PDZ domain containing family member 2	0.1
273	MKS1	MKS transition zone complex subunit 1	0.1
274	CRTAC1	cartilage acidic protein 1	0.1
275	PPP2R1B	protein phosphatase 2 scaffold subunit Abeta	0.1
276	CARMIL1	capping protein regulator and myosin 1 linker 1	0.1
277	AXL	AXL receptor tyrosine kinase	0.1
278	APOM	apolipoprotein M	0.1
279	MAPK1	mitogen-activated protein kinase 1	0.1
280	MAPK3	mitogen-activated protein kinase 3	0.1
281	MAPK8	mitogen-activated protein kinase 8	0.1
282	MAP2K7	mitogen-activated protein kinase kinase 7	0.1
283	PSG2	pregnancy specific beta-1-glycoprotein 2	0.1
284	SLC12A9	solute carrier family 12 member 9	0.1
285	PTGS2	prostaglandin-endoperoxide synthase 2	0.1
286	PTHLH	parathyroid hormone like hormone	0.1
287	AHRR	aryl-hydrocarbon receptor repressor	0.1
288	LRFN2	leucine rich repeat and fibronectin type III domain containing 2	0.1
289	STAMBPL1	STAM binding protein like 1	0.1
290	PXN	paxillin	0.1
291	RAC1	Rac family small GTPase 1	0.1
292	RAD51	RAD51 recombinase	0.1
293	RAD51B	RAD51 paralog B	0.1
294	RAF1	Raf-1 proto-oncogene, serine/threonine kinase	0.1
295	ACTA2	actin alpha 2, smooth muscle	0.1
296	RARB	retinoic acid receptor beta	0.1
297	CCND1	cyclin D1	0.1
298	BCL2	BCL2 apoptosis regulator	0.1
299	RET	ret proto-oncogene	0.1
300	ACTB	actin beta	0.1
301	ABCE1	ATP binding cassette subfamily E member 1	0.1
302	ROS1	ROS proto-oncogene 1, receptor tyrosine kinase	0.1
303	RP1	RP1 axonemal microtubule associated	0.1
304	MIR486-1	microRNA 486-1	0.1
305	ATXN2	ataxin 2	0.1
306	CCL2	C-C motif chemokine ligand 2	0.1
307	CXCL12	C-X-C motif chemokine ligand 12	0.1
308	SEMA3F	semaphorin 3F	0.1
309	POU5F1P3	POU class 5 homeobox 1 pseudogene 3	0.1
310	LINC01829	long intergenic non-protein coding RNA 1829	0.1
311	POU5F1P4	POU class 5 homeobox 1 pseudogene 4	0.1
312	RPS18P9	ribosomal protein S18 pseudogene 9	0.1
313	SHOX2	short stature homeobox 2	0.1
314	BMI1	BMI1 proto-oncogene, polycomb ring finger	0.1
315	TMEM237	transmembrane protein 237	0.1

316	SLC2A1	solute carrier family 2 member 1	0.1
317	SMARCA4	SWI/SNF related, matrix associated, actin dependent regulator of chromatin, subfamily a, member 4	0.1
318	SOD2	superoxide dismutase 2	0.1
319	SOX2	SRY-box transcription factor 2	0.1
320	SOX9	SRY-box transcription factor 9	0.1
321	SP1	Sp1 transcription factor	0.1
322	SPP1	secreted phosphoprotein 1	0.1
323	STAT3	signal transducer and activator of transcription 3	0.1
324	STK11	serine/threonine kinase 11	0.1
325	HNF1B	HNF1 homeobox B	0.1
326	ZEB1	zinc finger E-box binding homeobox 1	0.1
327	TERT	telomerase reverse transcriptase	0.1
328	TGFB1	transforming growth factor beta 1	0.1
329	TIMP1	TIMP metallopeptidase inhibitor 1	0.1
330	NKX2-1	NK2 homeobox 1	0.1
331	TNF	tumor necrosis factor	0.1
332	TNXB	tenascin XB	0.1
333	TP73	tumor protein p73	0.1
334	TTF1	transcription termination factor 1	0.1
335	TWIST1	twist family bHLH transcription factor 1	0.1
336	TYMS	thymidylate synthetase	0.1
337	LINC01347	long intergenic non-protein coding RNA 1347	0.1
338	VDR	vitamin D receptor	0.1
339	VEGFA	vascular endothelial growth factor A	0.1
340	VEGFC	vascular endothelial growth factor C	0.1
341	VIM	vimentin	0.1
342	XPA	XPA, DNA damage recognition and repair factor	0.1
343	XPC	XPC complex subunit, DNA damage recognition and repair factor	0.1
344	XRCC1	X-ray repair cross complementing 1	0.1
345	XRCC3	X-ray repair cross complementing 3	0.1
346	TRIM26	tripartite motif containing 26	0.1
347	CXCR4	C-X-C motif chemokine receptor 4	0.1
348	SCLC1	small cell cancer of the lung	0.1
349	AIMP2	aminoacyl tRNA synthetase complex interacting multifunctional protein 2	0.1
350	SEMA6D	semaphorin 6D	0.1
351	SLC44A4	solute carrier family 44 member 4	0.1
352	HMGA2	high mobility group AT-hook 2	0.1
353	H2AC6	H2A clustered histone 6	0.1
354	ARHGAP24	Rho GTPase activating protein 24	0.1
355	CASP3	caspase 3	0.1
356	USHBP1	USH1 protein network component harmonin binding protein 1	0.1
357	CAT	catalase	0.1
358	TUBA1C	tubulin alpha 1c	0.1
359	ATAD1	ATPase family AAA domain containing 1	0.1
360	CAV1	caveolin 1	0.1
361	BECN1	beclin 1	0.1

362	DNAH11	dynein axonemal heavy chain 11	0.1
363	ABCC3	ATP binding cassette subfamily C member 3	0.1
364	TNFSF10	TNF superfamily member 10	0.1
365	PROM1	prominin 1	0.1
366	HSPB3	heat shock protein family B (small) member 3	0.1
367	ACVR1B	activin A receptor type 1B	0.1
368	SYNGR2	synaptogyrin 2	0.1
369	TMEM132C	transmembrane protein 132C	0.1
370	NUMBL	NUMB like endocytic adaptor protein	0.1
371	KLF4	Kruppel like factor 4	0.1
372	GRAP2	GRB2 related adaptor protein 2	0.1
373	MUC16	mucin 16, cell surface associated	0.1
374	ABCG2	ATP binding cassette subfamily G member 2 (Junior blood group)	0.1
375	CD44	CD44 molecule (Indian blood group)	0.1
376	SECISBP2L	SECIS binding protein 2 like	0.1
377	KEAP1	kelch like ECH associated protein 1	0.1
378	CEP170	centrosomal protein 170	0.1
379	MVP	major vault protein	0.1
380	CDH1	cadherin 1	0.1

Table S5: Lung Cancer associated genes extracted from the TCGA database with Single Somatic Mutation (SSM) percentage ≥ 1

S.No.	Symbol	Name	# SSM Affected Cases in Cohort
1	TP53	tumor protein p53	687 / 1,040 (66.06%)
2	MUC16	mucin 16, cell surface associated	485 / 1,040 (46.63%)
3	CSMD3	CUB and Sushi multiple domains 3	463 / 1,040 (44.52%)
4	LRP1B	LDL receptor related protein 1B	386 / 1,040 (37.12%)
5	FAT3	FAT atypical cadherin 3	235 / 1,040 (22.60%)
6	FAM135B	family with sequence similarity 135 member B	225 / 1,040 (21.63%)
7	CDH10	cadherin 10	208 / 1,040 (20.00%)
8	FAT4	FAT atypical cadherin 4	184 / 1,040 (17.69%)
9	CNTNAP2	contactin associated protein 2	161 / 1,040 (15.48%)
10	KRAS	KRAS proto-oncogene, GTPase	157 / 1,040 (15.10%)
11	KMT2D	lysine methyltransferase 2D	154 / 1,040 (14.81%)
12	KEAP1	kelch like ECH associated protein 1	148 / 1,040 (14.23%)
13	CTNNA2	catenin alpha 2	145 / 1,040 (13.94%)
14	CTNND2	catenin delta 2	138 / 1,040 (13.27%)
15	PTPRD	protein tyrosine phosphatase receptor type D	138 / 1,040 (13.27%)
16	FAM47C	family with sequence similarity 47 member C	128 / 1,040 (12.31%)
17	FAT1	FAT atypical cadherin 1	124 / 1,040 (11.92%)
18	NF1	neurofibromin 1	118 / 1,040 (11.35%)
19	KMT2C	lysine methyltransferase 2C	117 / 1,040 (11.25%)
20	GRIN2A	glutamate ionotropic receptor NMDA type subunit 2A	112 / 1,040 (10.77%)
21	ZNF521	zinc finger protein 521	107 / 1,040 (10.29%)
22	BIRC6	baculoviral IAP repeat containing 6	106 / 1,040 (10.19%)
23	PTPRT	protein tyrosine phosphatase receptor type T	104 / 1,040 (10.00%)
24	COL3A1	collagen type III alpha 1 chain	101 / 1,040 (9.71%)
25	CDKN2A	cyclin dependent kinase inhibitor 2A	101 / 1,040 (9.71%)
26	PTPRB	protein tyrosine phosphatase receptor type B	101 / 1,040 (9.71%)
27	EPHA3	EPH receptor A3	100 / 1,040 (9.62%)
28	ERBB4	erb-b2 receptor tyrosine kinase 4	97 / 1,040 (9.33%)
29	SETBP1	SET binding protein 1	94 / 1,040 (9.04%)
30	DCC	DCC netrin 1 receptor	94 / 1,040 (9.04%)
31	ANK1	ankyrin 1	93 / 1,040 (8.94%)
32	ZNF479	zinc finger protein 479	93 / 1,040 (8.94%)
33	EGFR	epidermal growth factor receptor	93 / 1,040 (8.94%)
34	KDR	kinase insert domain receptor	89 / 1,040 (8.56%)
35	TRRAP	transformation/transcription domain associated protein	88 / 1,040 (8.46%)
36	PREX2	phosphatidylinositol-3,4,5-trisphosphate dependent Rac exchange factor 2	88 / 1,040 (8.46%)
37	NFE2L2	nuclear factor, erythroid 2 like 2	87 / 1,040 (8.37%)
38	NTRK3	neurotrophic receptor tyrosine kinase 3	86 / 1,040 (8.27%)
39	PIK3CA	phosphatidylinositol-4,5-bisphosphate 3-kinase catalytic subunit alpha	85 / 1,040 (8.17%)

40	ZFHX3	zinc finger homeobox 3	82 / 1,040 (7.88%)
41	PTPRC	protein tyrosine phosphatase receptor type C	80 / 1,040 (7.69%)
42	POLQ	DNA polymerase theta	79 / 1,040 (7.60%)
43	FLNA	filamin A	78 / 1,040 (7.50%)
44	ROBO2	roundabout guidance receptor 2	78 / 1,040 (7.50%)
45	ZEB1	zinc finger E-box binding homeobox 1	77 / 1,040 (7.40%)
46	TPR	translocated promoter region, nuclear basket protein	77 / 1,040 (7.40%)
47	AKAP9	A-kinase anchoring protein 9	76 / 1,040 (7.31%)
48	NBEA	neurobeachin	75 / 1,040 (7.21%)
49	ATM	ATM serine/threonine kinase	75 / 1,040 (7.21%)
50	RUNX1T1	RUNX1 partner transcriptional co-repressor 1	74 / 1,040 (7.12%)
51	RNF213	ring finger protein 213	72 / 1,040 (6.92%)
52	STK11	serine/threonine kinase 11	71 / 1,040 (6.83%)
53	CDH11	cadherin 11	71 / 1,040 (6.83%)
54	RGS7	regulator of G protein signaling 7	71 / 1,040 (6.83%)
55	PDGFRA	platelet derived growth factor receptor alpha	70 / 1,040 (6.73%)
56	RANBP2	RAN binding protein 2	70 / 1,040 (6.73%)
57	MYH11	myosin heavy chain 11	69 / 1,040 (6.63%)
58	ROS1	ROS proto-oncogene 1, receptor tyrosine kinase	69 / 1,040 (6.63%)
59	DCAF12L2	DDB1 and CUL4 associated factor 12 like 2	68 / 1,040 (6.54%)
60	CAMTA1	calmodulin binding transcription activator 1	68 / 1,040 (6.54%)
61	ARID1A	AT-rich interaction domain 1A	68 / 1,040 (6.54%)
62	MED12	mediator complex subunit 12	67 / 1,040 (6.44%)
63	KMT2A	lysine methyltransferase 2A	67 / 1,040 (6.44%)
64	BRCA2	BRCA2 DNA repair associated	67 / 1,040 (6.44%)
65	GRM3	glutamate metabotropic receptor 3	67 / 1,040 (6.44%)
66	RB1	RB transcriptional corepressor 1	66 / 1,040 (6.35%)
67	ATRX	ATRX chromatin remodeler	66 / 1,040 (6.35%)
68	CREBBP	CREB binding protein	65 / 1,040 (6.25%)
69	EPHA7	EPH receptor A7	65 / 1,040 (6.25%)
70	SMARCA4	SWI/SNF related, matrix associated, actin dependent regulator of chromatin, subfamily a, member 4	65 / 1,040 (6.25%)
71	PTEN	phosphatase and tensin homolog	65 / 1,040 (6.25%)
72	AMER1	APC membrane recruitment protein 1	65 / 1,040 (6.25%)
73	IL7R	interleukin 7 receptor	64 / 1,040 (6.15%)
74	APC	APC regulator of WNT signaling pathway	61 / 1,040 (5.87%)
75	POLE	DNA polymerase epsilon, catalytic subunit	61 / 1,040 (5.87%)
76	NOTCH1	notch receptor 1	61 / 1,040 (5.87%)
77	IRS4	insulin receptor substrate 4	60 / 1,040 (5.77%)
78	ALK	ALK receptor tyrosine kinase	60 / 1,040 (5.77%)
79	ARID1B	AT-rich interaction domain 1B	60 / 1,040 (5.77%)
80	UBR5	ubiquitin protein ligase E3 component n-recognin 5	60 / 1,040 (5.77%)
81	GPC5	glypican 5	58 / 1,040 (5.58%)
82	COL2A1	collagen type II alpha 1 chain	58 / 1,040 (5.58%)

83	BCORL1	BCL6 corepressor like 1	57 / 1,040 (5.48%)
84	ARID2	AT-rich interaction domain 2	57 / 1,040 (5.48%)
85	PDE4DIP	phosphodiesterase 4D interacting protein	55 / 1,040 (5.29%)
86	NCOR2	nuclear receptor corepressor 2	55 / 1,040 (5.29%)
87	ATP2B3	ATPase plasma membrane Ca2+ transporting 3	55 / 1,040 (5.29%)
88	PRKCB	protein kinase C beta	55 / 1,040 (5.29%)
89	KIAA1549	KIAA1549	55 / 1,040 (5.29%)
90	SETD2	SET domain containing 2, histone lysine methyltransferase	54 / 1,040 (5.19%)
91	MUC4	mucin 4, cell surface associated	54 / 1,040 (5.19%)
92	MTOR	mechanistic target of rapamycin kinase	54 / 1,040 (5.19%)
93	BRAF	B-Raf proto-oncogene, serine/threonine kinase	53 / 1,040 (5.10%)
94	MYH9	myosin heavy chain 9	53 / 1,040 (5.10%)
95	NSD1	nuclear receptor binding SET domain protein 1	52 / 1,040 (5.00%)
96	SPEN	spen family transcriptional repressor	51 / 1,040 (4.90%)
97	CARD11	caspase recruitment domain family member 11	51 / 1,040 (4.90%)
98	TET1	tet methylcytosine dioxygenase 1	50 / 1,040 (4.81%)
99	NOTCH2	notch receptor 2	49 / 1,040 (4.71%)
100	TNC	tenascin C	49 / 1,040 (4.71%)
101	ATR	ATR serine/threonine kinase	49 / 1,040 (4.71%)
102	BCL11A	BAF chromatin remodeling complex subunit BCL11A	46 / 1,040 (4.42%)
103	BRCA1	BRCA1 DNA repair associated	46 / 1,040 (4.42%)
104	FLT3	fms related receptor tyrosine kinase 3	45 / 1,040 (4.33%)
105	RET	ret proto-oncogene	45 / 1,040 (4.33%)
106	ASXL1	ASXL transcriptional regulator 1	44 / 1,040 (4.23%)
107	NUTM1	NUT midline carcinoma family member 1	44 / 1,040 (4.23%)
108	RBM10	RNA binding motif protein 10	44 / 1,040 (4.23%)
109	NCOR1	nuclear receptor corepressor 1	44 / 1,040 (4.23%)
110	SALL4	spalt like transcription factor 4	44 / 1,040 (4.23%)
111	PTPN13	protein tyrosine phosphatase non-receptor type 13	43 / 1,040 (4.13%)
112	NCOA2	nuclear receptor coactivator 2	43 / 1,040 (4.13%)
113	AFF3	AF4/FMR2 family member 3	43 / 1,040 (4.13%)
114	FBXW7	F-box and WD repeat domain containing 7	43 / 1,040 (4.13%)
115	GLI1	GLI family zinc finger 1	42 / 1,040 (4.04%)
116	KDM5C	lysine demethylase 5C	42 / 1,040 (4.04%)
117	PTPRK	protein tyrosine phosphatase receptor type K	42 / 1,040 (4.04%)
118	NCOA1	nuclear receptor coactivator 1	41 / 1,040 (3.94%)
119	NRG1	neuregulin 1	41 / 1,040 (3.94%)
120	DDR2	discoidin domain receptor tyrosine kinase 2	41 / 1,040 (3.94%)
121	LATS1	large tumor suppressor kinase 1	41 / 1,040 (3.94%)
122	BTK	Bruton tyrosine kinase	41 / 1,040 (3.94%)
123	FLT4	fms related receptor tyrosine kinase 4	40 / 1,040 (3.85%)
124	FCRL4	Fc receptor like 4	40 / 1,040 (3.85%)
125	CUX1	cut like homeobox 1	39 / 1,040 (3.75%)
126	NIN	ninein	39 / 1,040 (3.75%)

127	MLLT10	MLLT10 histone lysine methyltransferase DOT1L cofactor	39 / 1,040 (3.75%)
128	BCOR	BCL6 corepressor	39 / 1,040 (3.75%)
129	PRDM16	PR/SET domain 16	39 / 1,040 (3.75%)
130	ZMYM3	zinc finger MYM-type containing 3	39 / 1,040 (3.75%)
131	NTRK1	neurotrophic receptor tyrosine kinase 1	38 / 1,040 (3.65%)
132	ARHGEF1 2	Rho guanine nucleotide exchange factor 12	38 / 1,040 (3.65%)
133	TBX3	T-box transcription factor 3	38 / 1,040 (3.65%)
134	KDM6A	lysine demethylase 6A	38 / 1,040 (3.65%)
135	CDK12	cyclin dependent kinase 12	37 / 1,040 (3.56%)
136	CNBD1	cyclic nucleotide binding domain containing 1	37 / 1,040 (3.56%)
137	DICER1	dicer 1, ribonuclease III	37 / 1,040 (3.56%)
138	WT1	WT1 transcription factor	37 / 1,040 (3.56%)
139	AFDN	afadin, adherens junction formation factor	37 / 1,040 (3.56%)
140	DNMT3A	DNA methyltransferase 3 alpha	37 / 1,040 (3.56%)
141	KDM5A	lysine demethylase 5A	37 / 1,040 (3.56%)
142	ASXL2	ASXL transcriptional regulator 2	37 / 1,040 (3.56%)
143	A1CF	APOBEC1 complementation factor	37 / 1,040 (3.56%)
144	ABL2	ABL proto-oncogene 2, non-receptor tyrosine kinase	37 / 1,040 (3.56%)
145	NUP214	nucleoporin 214	37 / 1,040 (3.56%)
146	SRGAP3	SLIT-ROBO Rho GTPase activating protein 3	36 / 1,040 (3.46%)
147	TSHR	thyroid stimulating hormone receptor	36 / 1,040 (3.46%)
148	IL21R	interleukin 21 receptor	36 / 1,040 (3.46%)
149	ARHGAP 5	Rho GTPase activating protein 5	36 / 1,040 (3.46%)
150	USP6	ubiquitin specific peptidase 6	35 / 1,040 (3.37%)
151	STAG2	stromal antigen 2	35 / 1,040 (3.37%)
152	FLI1	Fli-1 proto-oncogene, ETS transcription factor	35 / 1,040 (3.37%)
153	CACNA1 D	calcium voltage-gated channel subunit alpha1 D	35 / 1,040 (3.37%)
154	KAT6A	lysine acetyltransferase 6A	35 / 1,040 (3.37%)
155	MYO5A	myosin VA	35 / 1,040 (3.37%)
156	NUP98	nucleoporin 98 and 96 precursor	35 / 1,040 (3.37%)
157	MB21D2	Mab-21 domain containing 2	34 / 1,040 (3.27%)
158	P2RY8	P2Y receptor family member 8	34 / 1,040 (3.27%)
159	CHD4	chromodomain helicase DNA binding protein 4	34 / 1,040 (3.27%)
160	COL1A1	collagen type I alpha 1 chain	34 / 1,040 (3.27%)
161	BCL9L	BCL9 like	34 / 1,040 (3.27%)
162	EP300	E1A binding protein p300	34 / 1,040 (3.27%)
163	KNL1	kinetochore scaffold 1	34 / 1,040 (3.27%)
164	CDH17	cadherin 17	34 / 1,040 (3.27%)
165	PRDM2	PR/SET domain 2	34 / 1,040 (3.27%)
166	MECOM	MDS1 and EVI1 complex locus	33 / 1,040 (3.17%)
167	SF3B1	splicing factor 3b subunit 1	33 / 1,040 (3.17%)
168	CSF1R	colony stimulating factor 1 receptor	33 / 1,040 (3.17%)
169	KIT	KIT proto-oncogene, receptor tyrosine kinase	33 / 1,040 (3.17%)

170	MET	MET proto-oncogene, receptor tyrosine kinase	33 / 1,040 (3.17%)
171	NFATC2	nuclear factor of activated T cells 2	33 / 1,040 (3.17%)
172	DROSHA	drosha ribonuclease III	33 / 1,040 (3.17%)
173	CNTRL	centriolin	33 / 1,040 (3.17%)
174	CUL3	cullin 3	33 / 1,040 (3.17%)
175	KAT6B	lysine acetyltransferase 6B	32 / 1,040 (3.08%)
176	EBF1	EBF transcription factor 1	32 / 1,040 (3.08%)
177	ACSL6	acyl-CoA synthetase long chain family member 6	32 / 1,040 (3.08%)
178	HIP1	huntingtin interacting protein 1	32 / 1,040 (3.08%)
179	GNAS	GNAS complex locus	32 / 1,040 (3.08%)
180	JAK2	Janus kinase 2	32 / 1,040 (3.08%)
181	STIL	STIL centriolar assembly protein	31 / 1,040 (2.98%)
182	IKZF1	IKAROS family zinc finger 1	31 / 1,040 (2.98%)
183	WRN	WRN RecQ like helicase	31 / 1,040 (2.98%)
184	ERC1	ELKS/RAB6-interacting/CAST family member 1	31 / 1,040 (2.98%)
185	TRIP11	thyroid hormone receptor interactor 11	30 / 1,040 (2.88%)
186	BRIP1	BRCA1 interacting protein C-terminal helicase 1	30 / 1,040 (2.88%)
187	SMAD4	SMAD family member 4	30 / 1,040 (2.88%)
188	MN1	MN1 proto-oncogene, transcriptional regulator	30 / 1,040 (2.88%)
189	GATA3	GATA binding protein 3	30 / 1,040 (2.88%)
190	LCP1	lymphocyte cytosolic protein 1	30 / 1,040 (2.88%)
191	PIK3CB	phosphatidylinositol-4,5-bisphosphate 3-kinase catalytic subunit beta	30 / 1,040 (2.88%)
192	TET2	tet methylcytosine dioxygenase 2	30 / 1,040 (2.88%)
193	CBLB	Cbl proto-oncogene B	30 / 1,040 (2.88%)
194	NSD3	nuclear receptor binding SET domain protein 3	30 / 1,040 (2.88%)
195	PTCH1	patched 1	29 / 1,040 (2.79%)
196	FOXP1	forkhead box P1	29 / 1,040 (2.79%)
197	FGFR2	fibroblast growth factor receptor 2	29 / 1,040 (2.79%)
198	NUMA1	nuclear mitotic apparatus protein 1	29 / 1,040 (2.79%)
199	SND1	staphylococcal nuclease and tudor domain containing 1	29 / 1,040 (2.79%)
200	MAML2	mastermind like transcriptional coactivator 2	29 / 1,040 (2.79%)
201	BCLAF1	BCL2 associated transcription factor 1	29 / 1,040 (2.79%)
202	TP63	tumor protein p63	29 / 1,040 (2.79%)
203	LIFR	LIF receptor subunit alpha	28 / 1,040 (2.69%)
204	TSC2	TSC complex subunit 2	28 / 1,040 (2.69%)
205	PAX3	paired box 3	28 / 1,040 (2.69%)
206	PALB2	partner and localizer of BRCA2	28 / 1,040 (2.69%)
207	SNX29	sorting nexin 29	28 / 1,040 (2.69%)
208	CLTC	clathrin heavy chain	28 / 1,040 (2.69%)
209	WNK2	WNK lysine deficient protein kinase 2	28 / 1,040 (2.69%)
210	ITK	IL2 inducible T cell kinase	28 / 1,040 (2.69%)
211	ESR1	estrogen receptor 1	28 / 1,040 (2.69%)
212	CLTCL1	clathrin heavy chain like 1	28 / 1,040 (2.69%)
213	DCTN1	dynactin subunit 1	28 / 1,040 (2.69%)

214	PDGFRB	platelet derived growth factor receptor beta	28 / 1,040 (2.69%)
215	PMS1	PMS1 homolog 1, mismatch repair system component	27 / 1,040 (2.60%)
216	BCR	BCR activator of RhoGEF and GTPase	27 / 1,040 (2.60%)
217	PRDM1	PR/SET domain 1	27 / 1,040 (2.60%)
218	VAV1	vav guanine nucleotide exchange factor 1	27 / 1,040 (2.60%)
219	LPP	LIM domain containing preferred translocation partner in lipoma	27 / 1,040 (2.60%)
220	POT1	protection of telomeres 1	27 / 1,040 (2.60%)
221	ZBTB16	zinc finger and BTB domain containing 16	27 / 1,040 (2.60%)
222	ZNF331	zinc finger protein 331	26 / 1,040 (2.50%)
223	MAP3K13	mitogen-activated protein kinase kinase kinase 13	26 / 1,040 (2.50%)
224	CYLD	CYLD lysine 63 deubiquitinase	26 / 1,040 (2.50%)
225	LARP4B	La ribonucleoprotein 4B	26 / 1,040 (2.50%)
226	N4BP2	NEDD4 binding protein 2	26 / 1,040 (2.50%)
227	ERBB2	erb-b2 receptor tyrosine kinase 2	26 / 1,040 (2.50%)
228	BAZ1A	bromodomain adjacent to zinc finger domain 1A	26 / 1,040 (2.50%)
229	LZTR1	leucine zipper like transcription regulator 1	26 / 1,040 (2.50%)
230	CIITA	class II major histocompatibility complex transactivator	26 / 1,040 (2.50%)
231	JAK3	Janus kinase 3	26 / 1,040 (2.50%)
232	GAS7	growth arrest specific 7	26 / 1,040 (2.50%)
233	BCL11B	BAF chromatin remodeling complex subunit BCL11B	25 / 1,040 (2.40%)
234	RBM15	RNA binding motif protein 15	25 / 1,040 (2.40%)
235	CSF3R	colony stimulating factor 3 receptor	25 / 1,040 (2.40%)
236	NACA	nascent polypeptide associated complex subunit alpha	25 / 1,040 (2.40%)
237	IGF2BP2	insulin like growth factor 2 mRNA binding protein 2	25 / 1,040 (2.40%)
238	BARD1	BRCA1 associated RING domain 1	25 / 1,040 (2.40%)
239	ITGAV	integrin subunit alpha V	24 / 1,040 (2.31%)
240	ERBB3	erb-b2 receptor tyrosine kinase 3	24 / 1,040 (2.31%)
241	MYB	MYB proto-oncogene, transcription factor	24 / 1,040 (2.31%)
242	FH	fumarate hydratase	24 / 1,040 (2.31%)
243	CLIP1	CAP-Gly domain containing linker protein 1	24 / 1,040 (2.31%)
244	ARHGEF10L	Rho guanine nucleotide exchange factor 10 like	24 / 1,040 (2.31%)
245	PPP2R1A	protein phosphatase 2 scaffold subunit Aalpha	24 / 1,040 (2.31%)
246	ABL1	ABL proto-oncogene 1, non-receptor tyrosine kinase	23 / 1,040 (2.21%)
247	SETDB1	SET domain bifurcated histone lysine methyltransferase 1	23 / 1,040 (2.21%)
248	KTN1	kinectin 1	23 / 1,040 (2.21%)
249	PER1	period circadian regulator 1	23 / 1,040 (2.21%)
250	ARHGEF10	Rho guanine nucleotide exchange factor 10	23 / 1,040 (2.21%)
251	SLC34A2	solute carrier family 34 member 2	23 / 1,040 (2.21%)
252	TEC	transient erythroblastopenia of childhood	23 / 1,040 (2.21%)
253	FAM131B	family with sequence similarity 131 member B	23 / 1,040 (2.21%)
254	CYSLTR2	cysteinyl leukotriene receptor 2	23 / 1,040 (2.21%)
255	AR	androgen receptor	23 / 1,040 (2.21%)
256	CTNNB1	catenin beta 1	23 / 1,040 (2.21%)

257	FGFR4	fibroblast growth factor receptor 4	23 / 1,040 (2.21%)
258	LRIG3	leucine rich repeats and immunoglobulin like domains 3	23 / 1,040 (2.21%)
259	TRIM24	tripartite motif containing 24	22 / 1,040 (2.12%)
260	PBRM1	polybromo 1	22 / 1,040 (2.12%)
261	STRN	striatin	22 / 1,040 (2.12%)
262	PAX7	paired box 7	22 / 1,040 (2.12%)
263	KLK2	kallikrein related peptidase 2	22 / 1,040 (2.12%)
264	ECT2L	epithelial cell transforming 2 like	22 / 1,040 (2.12%)
265	THRAP3	thyroid hormone receptor associated protein 3	22 / 1,040 (2.12%)
266	GPC3	glypican 3	22 / 1,040 (2.12%)
267	BCL9	BCL9 transcription coactivator	22 / 1,040 (2.12%)
268	PBX1	PBX homeobox 1	22 / 1,040 (2.12%)
269	H3C2	H3 clustered histone 2	22 / 1,040 (2.12%)
270	BMP5	bone morphogenetic protein 5	22 / 1,040 (2.12%)
271	BCL6	BCL6 transcription repressor	21 / 1,040 (2.02%)
272	FLCN	folliculin	21 / 1,040 (2.02%)
273	PML	PML nuclear body scaffold	21 / 1,040 (2.02%)
274	CHD2	chromodomain helicase DNA binding protein 2	21 / 1,040 (2.02%)
275	SMO	smoothened, frizzled class receptor	21 / 1,040 (2.02%)
276	AFF1	AF4/FMR2 family member 1	21 / 1,040 (2.02%)
277	JAK1	Janus kinase 1	21 / 1,040 (2.02%)
278	AFF4	AF4/FMR2 family member 4	21 / 1,040 (2.02%)
279	IKBKB	inhibitor of nuclear factor kappa B kinase subunit beta	21 / 1,040 (2.02%)
280	LEF1	lymphoid enhancer binding factor 1	21 / 1,040 (2.02%)
281	PRRX1	paired related homeobox 1	21 / 1,040 (2.02%)
282	MAP3K1	mitogen-activated protein kinase kinase kinase 1	21 / 1,040 (2.02%)
283	CTNND1	catenin delta 1	20 / 1,040 (1.92%)
284	MALT1	MALT1 paracaspase	20 / 1,040 (1.92%)
285	CRNKL1	crooked neck pre-mRNA splicing factor 1	20 / 1,040 (1.92%)
286	TGFBR2	transforming growth factor beta receptor 2	20 / 1,040 (1.92%)
287	EZH2	enhancer of zeste 2 polycomb repressive complex 2 subunit	20 / 1,040 (1.92%)
288	MLLT6	MLLT6, PHD finger containing	20 / 1,040 (1.92%)
289	FBLN2	fibulin 2	20 / 1,040 (1.92%)
290	DGCR8	DGCR8 microprocessor complex subunit	20 / 1,040 (1.92%)
291	PIK3R1	phosphoinositide-3-kinase regulatory subunit 1	20 / 1,040 (1.92%)
292	EPAS1	endothelial PAS domain protein 1	20 / 1,040 (1.92%)
293	SFRP4	secreted frizzled related protein 4	20 / 1,040 (1.92%)
294	TNFAIP3	TNF alpha induced protein 3	20 / 1,040 (1.92%)
295	USP8	ubiquitin specific peptidase 8	20 / 1,040 (1.92%)
296	TAL1	TAL bHLH transcription factor 1, erythroid differentiation factor	20 / 1,040 (1.92%)
297	B2M	beta-2-microglobulin	20 / 1,040 (1.92%)
298	PTPN11	protein tyrosine phosphatase non-receptor type 11	20 / 1,040 (1.92%)
299	DDX10	DEAD-box helicase 10	19 / 1,040 (1.83%)

300	PRF1	perforin 1	19 / 1,040 (1.83%)
301	TRIM27	tripartite motif containing 27	19 / 1,040 (1.83%)
302	CEP89	centrosomal protein 89	19 / 1,040 (1.83%)
303	TSC1	TSC complex subunit 1	19 / 1,040 (1.83%)
304	KCNJ5	potassium inwardly rectifying channel subfamily J member 5	19 / 1,040 (1.83%)
305	NSD2	nuclear receptor binding SET domain protein 2	19 / 1,040 (1.83%)
306	FCGR2B	Fc fragment of IgG receptor IIb	19 / 1,040 (1.83%)
307	FIP1L1	factor interacting with PAPOLA and CPSF1	19 / 1,040 (1.83%)
308	POLG	DNA polymerase gamma, catalytic subunit	19 / 1,040 (1.83%)
309	STAG1	stromal antigen 1	19 / 1,040 (1.83%)
310	PAX5	paired box 5	19 / 1,040 (1.83%)
311	WAS	WASP actin nucleation promoting factor	19 / 1,040 (1.83%)
312	SIRPA	signal regulatory protein alpha	19 / 1,040 (1.83%)
313	BLM	BLM RecQ like helicase	19 / 1,040 (1.83%)
314	MACC1	MET transcriptional regulator MACC1	19 / 1,040 (1.83%)
315	TRIM33	tripartite motif containing 33	19 / 1,040 (1.83%)
316	DAXX	death domain associated protein	18 / 1,040 (1.73%)
317	BRD4	bromodomain containing 4	18 / 1,040 (1.73%)
318	FBXO11	F-box protein 11	18 / 1,040 (1.73%)
319	BUB1B	BUB1 mitotic checkpoint serine/threonine kinase B	18 / 1,040 (1.73%)
320	MSH6	mutS homolog 6	18 / 1,040 (1.73%)
321	MAP2K1	mitogen-activated protein kinase kinase 1	18 / 1,040 (1.73%)
322	PPFIBP1	PPFIA binding protein 1	18 / 1,040 (1.73%)
323	NR4A3	nuclear receptor subfamily 4 group A member 3	18 / 1,040 (1.73%)
324	FGFR1	fibroblast growth factor receptor 1	18 / 1,040 (1.73%)
325	LATS2	large tumor suppressor kinase 2	18 / 1,040 (1.73%)
326	EIF3E	eukaryotic translation initiation factor 3 subunit E	18 / 1,040 (1.73%)
327	GATA1	GATA binding protein 1	18 / 1,040 (1.73%)
328	CDC73	cell division cycle 73	18 / 1,040 (1.73%)
329	GMPS	guanine monophosphate synthase	18 / 1,040 (1.73%)
330	CBL	Cbl proto-oncogene	18 / 1,040 (1.73%)
331	AXIN1	axin 1	18 / 1,040 (1.73%)
332	TERT	telomerase reverse transcriptase	18 / 1,040 (1.73%)
333	RSPO2	R-spondin 2	17 / 1,040 (1.63%)
334	RAD21	RAD21 cohesin complex component	17 / 1,040 (1.63%)
335	GPHN	gephyrin	17 / 1,040 (1.63%)
336	PLAG1	PLAG1 zinc finger	17 / 1,040 (1.63%)
337	EXT1	exostosin glycosyltransferase 1	17 / 1,040 (1.63%)
338	PCM1	pericentriolar material 1	17 / 1,040 (1.63%)
339	CASP8	caspase 8	17 / 1,040 (1.63%)
340	IRF4	interferon regulatory factor 4	17 / 1,040 (1.63%)
341	STAT3	signal transducer and activator of transcription 3	17 / 1,040 (1.63%)
342	MSH2	mutS homolog 2	17 / 1,040 (1.63%)
343	ISX	intestine specific homeobox	17 / 1,040 (1.63%)

344	SMC1A	structural maintenance of chromosomes 1A	17 / 1,040 (1.63%)
345	FOXO4	forkhead box O4	17 / 1,040 (1.63%)
346	PRPF40B	pre-mRNA processing factor 40 homolog B	17 / 1,040 (1.63%)
347	MSN	moesin	17 / 1,040 (1.63%)
348	NBN	nibrin	17 / 1,040 (1.63%)
349	HOOK3	hook microtubule tethering protein 3	17 / 1,040 (1.63%)
350	CD209	CD209 molecule	17 / 1,040 (1.63%)
351	DDX3X	DEAD-box helicase 3 X-linked	17 / 1,040 (1.63%)
352	RHOH	ras homolog family member H	16 / 1,040 (1.54%)
353	FGFR3	fibroblast growth factor receptor 3	16 / 1,040 (1.54%)
354	TBL1XR1	TBL1X receptor 1	16 / 1,040 (1.54%)
355	ELN	elastin	16 / 1,040 (1.54%)
356	MRTFA	myocardin related transcription factor A	16 / 1,040 (1.54%)
357	PPARG	peroxisome proliferator activated receptor gamma	16 / 1,040 (1.54%)
358	LCK	LCK proto-oncogene, Src family tyrosine kinase	16 / 1,040 (1.54%)
359	TOP1	DNA topoisomerase I	16 / 1,040 (1.54%)
360	HSP90AB1	heat shock protein 90 alpha family class B member 1	16 / 1,040 (1.54%)
361	SGK1	serum/glucocorticoid regulated kinase 1	16 / 1,040 (1.54%)
362	ETV6	ETS variant transcription factor 6	16 / 1,040 (1.54%)
363	CIC	capicua transcriptional repressor	16 / 1,040 (1.54%)
364	EIF4A2	eukaryotic translation initiation factor 4A2	16 / 1,040 (1.54%)
365	PAX8	paired box 8	16 / 1,040 (1.54%)
366	TCF12	transcription factor 12	16 / 1,040 (1.54%)
367	EXT2	exostosin glycosyltransferase 2	16 / 1,040 (1.54%)
368	FUBP1	far upstream element binding protein 1	16 / 1,040 (1.54%)
369	MLH1	mutL homolog 1	16 / 1,040 (1.54%)
370	CRLF2	cytokine receptor like factor 2	16 / 1,040 (1.54%)
371	CBFA2T3	CBFA2/RUNX1 partner transcriptional co-repressor 3	15 / 1,040 (1.44%)
372	POLD1	DNA polymerase delta 1, catalytic subunit	15 / 1,040 (1.44%)
373	ATIC	5-aminoimidazole-4-carboxamide ribonucleotide formyltransferase/IMP cyclohydrolase	15 / 1,040 (1.44%)
374	PHOX2B	paired like homeobox 2B	15 / 1,040 (1.44%)
375	CXCR4	C-X-C motif chemokine receptor 4	15 / 1,040 (1.44%)
376	PMS2	PMS1 homolog 2, mismatch repair system component	15 / 1,040 (1.44%)
377	CREB3L2	cAMP responsive element binding protein 3 like 2	15 / 1,040 (1.44%)
378	GOLGA5	golgin A5	15 / 1,040 (1.44%)
379	NFIB	nuclear factor I B	15 / 1,040 (1.44%)
380	SDHA	succinate dehydrogenase complex flavoprotein subunit A	15 / 1,040 (1.44%)
381	CTCF	CCCTC-binding factor	15 / 1,040 (1.44%)
382	ERCC4	ERCC excision repair 4, endonuclease catalytic subunit	15 / 1,040 (1.44%)
383	CDH1	cadherin 1	15 / 1,040 (1.44%)
384	HOXC11	homeobox C11	15 / 1,040 (1.44%)
385	AKT2	AKT serine/threonine kinase 2	15 / 1,040 (1.44%)
386	HOXD13	homeobox D13	15 / 1,040 (1.44%)
387	RNF43	ring finger protein 43	15 / 1,040 (1.44%)

388	HNF1A	HNF1 homeobox A	15 / 1,040 (1.44%)
389	AKT3	AKT serine/threonine kinase 3	15 / 1,040 (1.44%)
390	CARS1	cysteinyl-tRNA synthetase 1	15 / 1,040 (1.44%)
391	QKI	QKI, KH domain containing RNA binding	15 / 1,040 (1.44%)
392	CNOT3	CCR4-NOT transcription complex subunit 3	15 / 1,040 (1.44%)
393	SEPTIN6	septin 6	15 / 1,040 (1.44%)
394	GATA2	GATA binding protein 2	15 / 1,040 (1.44%)
395	FANCA	FA complementation group A	15 / 1,040 (1.44%)
396	RPL10	ribosomal protein L10	15 / 1,040 (1.44%)
397	KAT7	lysine acetyltransferase 7	15 / 1,040 (1.44%)
398	PATZ1	POZ/BTB and AT hook containing zinc finger 1	14 / 1,040 (1.35%)
399	ETV5	ETS variant transcription factor 5	14 / 1,040 (1.35%)
400	ZMYM2	zinc finger MYM-type containing 2	14 / 1,040 (1.35%)
401	MYCN	MYCN proto-oncogene, bHLH transcription factor	14 / 1,040 (1.35%)
402	RFWD3	ring finger and WD repeat domain 3	14 / 1,040 (1.35%)
403	SS18	SS18 subunit of BAF chromatin remodeling complex	14 / 1,040 (1.35%)
404	SIX2	SIX homeobox 2	14 / 1,040 (1.35%)
405	MLLT3	MLLT3 super elongation complex subunit	14 / 1,040 (1.35%)
406	MYOD1	myogenic differentiation 1	14 / 1,040 (1.35%)
407	CDX2	caudal type homeobox 2	14 / 1,040 (1.35%)
408	TFE3	transcription factor binding to IGHM enhancer 3	14 / 1,040 (1.35%)
409	HLF	HLF transcription factor, PAR bZIP family member	14 / 1,040 (1.35%)
410	ERG	ETS transcription factor ERG	14 / 1,040 (1.35%)
411	TCF7L2	transcription factor 7 like 2	14 / 1,040 (1.35%)
412	MAP2K4	mitogen-activated protein kinase kinase 4	14 / 1,040 (1.35%)
413	ACVR1	activin A receptor type 1	14 / 1,040 (1.35%)
414	FANCD2	FA complementation group D2	14 / 1,040 (1.35%)
415	XPO1	exportin 1	14 / 1,040 (1.35%)
416	ACKR3	atypical chemokine receptor 3	14 / 1,040 (1.35%)
417	WIF1	WNT inhibitory factor 1	14 / 1,040 (1.35%)
418	KIF5B	kinesin family member 5B	14 / 1,040 (1.35%)
419	HOXA13	homeobox A13	14 / 1,040 (1.35%)
420	EPS15	epidermal growth factor receptor pathway substrate 15	14 / 1,040 (1.35%)
421	MITF	melanocyte inducing transcription factor	14 / 1,040 (1.35%)
422	STAT5B	signal transducer and activator of transcription 5B	14 / 1,040 (1.35%)
423	EML4	EMAP like 4	13 / 1,040 (1.25%)
424	S100A7	S100 calcium binding protein A7	13 / 1,040 (1.25%)
425	SPECC1	sperm antigen with calponin homology and coiled-coil domains 1	13 / 1,040 (1.25%)
426	BAP1	BRCA1 associated protein 1	13 / 1,040 (1.25%)
427	SMAD3	SMAD family member 3	13 / 1,040 (1.25%)
428	AXIN2	axin 2	13 / 1,040 (1.25%)
429	SMAD2	SMAD family member 2	13 / 1,040 (1.25%)
430	PLCG1	phospholipase C gamma 1	13 / 1,040 (1.25%)
431	ARNT	aryl hydrocarbon receptor nuclear translocator	13 / 1,040 (1.25%)

432	HLA-A	major histocompatibility complex, class I, A	13 / 1,040 (1.25%)
433	RAP1GDS1	Rap1 GTPase-GDP dissociation stimulator 1	13 / 1,040 (1.25%)
434	PICALM	phosphatidylinositol binding clathrin assembly protein	13 / 1,040 (1.25%)
435	SIX1	SIX homeobox 1	13 / 1,040 (1.25%)
436	ZNF429	zinc finger protein 429	13 / 1,040 (1.25%)
437	STAT6	signal transducer and activator of transcription 6	13 / 1,040 (1.25%)
438	HNRNPA2B1	heterogeneous nuclear ribonucleoprotein A2/B1	13 / 1,040 (1.25%)
439	CHEK2	checkpoint kinase 2	13 / 1,040 (1.25%)
440	RARA	retinoic acid receptor alpha	13 / 1,040 (1.25%)
441	PSIP1	PC4 and SFRS1 interacting protein 1	13 / 1,040 (1.25%)
442	H3-3B	H3.3 histone B	13 / 1,040 (1.25%)
443	LSM14A	LSM14A mRNA processing body assembly factor	12 / 1,040 (1.15%)
444	ACVR2A	activin A receptor type 2A	12 / 1,040 (1.15%)
445	MSI2	musashi RNA binding protein 2	12 / 1,040 (1.15%)
446	BMPR1A	bone morphogenetic protein receptor type 1A	12 / 1,040 (1.15%)
447	EWSR1	EWS RNA binding protein 1	12 / 1,040 (1.15%)
448	ZNRF3	zinc and ring finger 3	12 / 1,040 (1.15%)
449	RPL5	ribosomal protein L5	12 / 1,040 (1.15%)
450	CYP2C8	cytochrome P450 family 2 subfamily C member 8	12 / 1,040 (1.15%)
451	FOXL2	forkhead box L2	12 / 1,040 (1.15%)
452	ARHGAP26	Rho GTPase activating protein 26	12 / 1,040 (1.15%)
453	ELF4	E74 like ETS transcription factor 4	12 / 1,040 (1.15%)
454	BIRC3	baculoviral IAP repeat containing 3	12 / 1,040 (1.15%)
455	RECQL4	RecQ like helicase 4	12 / 1,040 (1.15%)
456	HIF1A	hypoxia inducible factor 1 subunit alpha	12 / 1,040 (1.15%)
457	ASPSCR1	ASPSCR1 tether for SLC2A4, UBX domain containing	12 / 1,040 (1.15%)
458	TCL1A	TCL1 family AKT coactivator A	12 / 1,040 (1.15%)
459	NCKIPSD	NCK interacting protein with SH3 domain	12 / 1,040 (1.15%)
460	SEPTIN9	septin 9	12 / 1,040 (1.15%)
461	ERCC3	ERCC excision repair 3, TFIIH core complex helicase subunit	12 / 1,040 (1.15%)
462	ZCCHC8	zinc finger CCHC-type containing 8	12 / 1,040 (1.15%)
463	CASP9	caspase 9	12 / 1,040 (1.15%)
464	NFKB2	nuclear factor kappa B subunit 2	11 / 1,040 (1.06%)
465	MUTYH	mutY DNA glycosylase	11 / 1,040 (1.06%)
466	ZNF384	zinc finger protein 384	11 / 1,040 (1.06%)
467	HSP90AA1	heat shock protein 90 alpha family class A member 1	11 / 1,040 (1.06%)
468	RAD17	RAD17 checkpoint clamp loader component	11 / 1,040 (1.06%)
469	FKBP9	FKBP prolyl isomerase 9	11 / 1,040 (1.06%)
470	XPC	XPC complex subunit, DNA damage recognition and repair factor	11 / 1,040 (1.06%)
471	ETNK1	ethanolamine kinase 1	11 / 1,040 (1.06%)
472	SHTN1	shootin 1	11 / 1,040 (1.06%)

473	NRAS	NRAS proto-oncogene, GTPase	11 / 1,040 (1.06%)
474	FANCF	FA complementation group F	11 / 1,040 (1.06%)
475	MAX	MYC associated factor X	11 / 1,040 (1.06%)
476	NF2	neurofibromin 2	11 / 1,040 (1.06%)
477	TRAF7	TNF receptor associated factor 7	11 / 1,040 (1.06%)
478	WDCP	WD repeat and coiled coil containing	11 / 1,040 (1.06%)
479	KNSTRN	kinetochore localized astrin (SPAG5) binding protein	11 / 1,040 (1.06%)
480	REL	REL proto-oncogene, NF-kB subunit	11 / 1,040 (1.06%)
481	ETV1	ETS variant transcription factor 1	11 / 1,040 (1.06%)
482	FOXO3	forkhead box O3	11 / 1,040 (1.06%)
483	PPM1D	protein phosphatase, Mg2+/Mn2+ dependent 1D	11 / 1,040 (1.06%)
484	SEPTIN5	septin 5	11 / 1,040 (1.06%)
485	FANCG	FA complementation group G	11 / 1,040 (1.06%)
486	SLC45A3	solute carrier family 45 member 3	11 / 1,040 (1.06%)
487	HOXA9	homeobox A9	11 / 1,040 (1.06%)
488	CHST11	carbohydrate sulfotransferase 11	11 / 1,040 (1.06%)
489	TAF15	TATA-box binding protein associated factor 15	11 / 1,040 (1.06%)
490	SMARCD1	SWI/SNF related, matrix associated, actin dependent regulator of chromatin, subfamily d, member 1	11 / 1,040 (1.06%)
491	CCNE1	cyclin E1	11 / 1,040 (1.06%)
492	DNM2	dynamin 2	11 / 1,040 (1.06%)

Table S6: Lung Cancer-associated proteins and targets of M. oleifera, bioactive compounds there were 80 intersecting proteins among them.

S.No.	Protein	Description
1	PIK3CA	Phosphatidylinositol-4,5-Bisphosphate 3-Kinase Catalytic Subunit Alpha
2	EGFR	Epidermal Growth Factor Receptor
3	ERBB2	Erb-B2 Receptor Tyrosine Kinase 2
4	DNMT1	DNA Methyltransferase 1
5	CTNNB1	Catenin Beta 1
6	DNMT3A	DNA Methyltransferase 3 Alpha
7	CHRNB4	Cholinergic Receptor Nicotinic Beta 4 Subunit
8	CHRNA3	Cholinergic Receptor Nicotinic Alpha 3 Subunit
9	CYP2A6	Cytochrome P450 Family 2 Subfamily A Member 6
10	STAT3	Signal Transducer And Activator Of Transcription 3
11	PIK3CD	Phosphatidylinositol-4,5-Bisphosphate 3-Kinase Catalytic Subunit Delta
12	FGFR1	Fibroblast Growth Factor Receptor 1
13	RARB	Retinoic Acid Receptor Beta
14	MDM2	MDM2 Proto-Oncogene
15	PIK3CB	Phosphatidylinositol-4,5-Bisphosphate 3-Kinase Catalytic Subunit Beta
16	ESR1	Estrogen Receptor 1
17	MAPK8	Mitogen-Activated Protein Kinase 8
18	MAPK1	Mitogen-Activated Protein Kinase 1
19	PTGS2	Prostaglandin-Endoperoxide Synthase 2
20	SLC2A1	Solute Carrier Family 2 Member 1
21	TERT	Telomerase Reverse Transcriptase
22	MET	MET Proto-Oncogene, Receptor Tyrosine Kinase
23	IGF1R	Insulin Like Growth Factor 1 Receptor
24	MCL1	MCL1 Apoptosis Regulator, BCL2 Family Member
25	ALDH1A1	Aldehyde Dehydrogenase 1 Family Member A1
26	IGFBP3	Insulin Like Growth Factor Binding Protein 3
27	CCND1	Cyclin D1
28	HSP90AA1	Heat Shock Protein 90 Alpha Family Class A Member 1
29	MMP2	Matrix Metallopeptidase 2
30	TYMS	Thymidylate Synthetase
31	JAK2	Janus Kinase 2
32	ALB	Albumin
33	HDAC1	Histone Deacetylase 1
34	CDK2	Cyclin Dependent Kinase 2
35	MAPK14	Mitogen-Activated Protein Kinase 14
36	IL1B	Interleukin 1 Beta
37	CASP3	Caspase 3
38	HRAS	HRas Proto-Oncogene, GTPase
39	CDK4	Cyclin Dependent Kinase 4
40	SERPINE1	Serpin Family E Member 1

41	MAPK3	Mitogen-Activated Protein Kinase 3
42	ICAM1	Intercellular Adhesion Molecule 1
43	FGF2	Fibroblast Growth Factor 2
44	GSTP1	Glutathione S-Transferase Pi 1
45	NFE2L2	NFE2 Like BZIP Transcription Factor 2
46	GSTM1	Glutathione S-Transferase Mu 1
47	ELANE	Elastase, Neutrophil Expressed
48	PARP1	Poly(ADP-Ribose) Polymerase 1
49	CHRNA4	Cholinergic Receptor Nicotinic Alpha 4 Subunit
50	VEGFA	Vascular Endothelial Growth Factor A
51	CYP1B1	Cytochrome P450 Family 1 Subfamily B Member 1
52	ABCB1	ATP Binding Cassette Subfamily B Member 1
53	PIK3CG	Phosphatidylinositol-4,5-Bisphosphate 3-Kinase Catalytic Subunit Gamma
54	GSTM2	Glutathione S-Transferase Mu 2
55	ESR2	Estrogen Receptor 2
56	EPHX1	Epoxide Hydrolase 1
57	MMP7	Matrix Metallopeptidase 7
58	CYP1A2	Cytochrome P450 Family 1 Subfamily A Member 2
59	MPO	Myeloperoxidase
60	MMP9	Matrix Metallopeptidase 9
61	CYP1A1	Cytochrome P450 Family 1 Subfamily A Member 1
62	MMP13	Matrix Metallopeptidase 13
63	MMP1	Matrix Metallopeptidase 1
64	CYP2D6	Cytochrome P450 Family 2 Subfamily D Member 6
65	ACE	Angiotensin I Converting Enzyme
66	KDR	Kinase Insert Domain Receptor
67	PTPRC	Protein Tyrosine Phosphatase Receptor Type C
68	PDGFRA	Platelet Derived Growth Factor Receptor Alpha
69	CREBBP	CREB Binding Protein
70	FLT3	Fms Related Receptor Tyrosine Kinase 3
71	KDM5C	Lysine Demethylase 5C
72	KDM5A	Lysine Demethylase 5A
73	EP300	E1A Binding Protein P300
74	KIT	KIT Proto-Oncogene, Receptor Tyrosine Kinase
75	PDGFRB	Platelet Derived Growth Factor Receptor Beta
76	JAK3	Janus Kinase 3
77	ABL1	ABL Proto-Oncogene 1, Non-Receptor Tyrosine Kinase
78	AR	Androgen Receptor
79	JAK1	Janus Kinase 1
80	IKBKB	Inhibitor Of Nuclear Factor Kappa B Kinase Subunit Beta

*Table S7: Table illustrate the information about topology parameters which is obtained after analysing the network of intersection proteins. The 11 key proteins which are selected on the median cutoff criteria: a degree value of ≥6, a CC value of ≥0.0295, and a BC value of ≥0.4093 are marked with symbol **(#)**.*

Proteins	Degree	ClosenessCentrality	BetweennessCentrality
STAT3#	26	0.530201342	0.357641768
PIK3R1	21	0.427027027	0.024045939
PIK3CA	21	0.422459893	0.021755018
EGFR#	20	0.443820225	0.037981457
ESR1#	19	0.473053892	0.105973467
PIK3CB	19	0.389162562	0.008878713
PIK3CD	19	0.389162562	0.008878713
HRAS#	18	0.413612565	0.076845333
JAK2#	18	0.409326425	0.076726529
CTNNB1#	16	0.422459893	0.066676348
IGF1R#	16	0.427027027	0.03278539
ERBB2	16	0.429347826	0.019354144
PLCG1	15	0.357466063	0.011216894
JAK1	14	0.398989899	0.002153115
HDAC1	13	0.407216495	0.088954975
PDGFRB	13	0.407216495	0.006862838
HSP90AA1#	12	0.415789474	0.067519845
CREBBP#	12	0.420212766	0.044162311
EP300#	12	0.409326425	0.041212571
KDR	12	0.387254902	0.028859898
PDGFRA	12	0.403061224	0.005733546
MET	11	0.409326425	0.008821869
MAPK1	10	0.407216495	0.02944081
CCND1	10	0.405128205	0.024650259
AR	10	0.405128205	0.010777391
MAPK8#	9	0.436464088	0.245525882
CYP1A1	9	0.25732899	0.030098453
FGFR1	9	0.393034826	0.014175791
MDM2	9	0.391089109	0.010557098
JAK3	9	0.385365854	6.98E-04
CYP2A6	8	0.256493506	0.029557503
FGF2	8	0.343478261	0.02876409
CYP1A2	8	0.256493506	0.017440225
MAPK3	8	0.360730594	0.007764452
MMP9	7	0.301526718	0.05665808
CASP3	7	0.36744186	0.041431254
KIT	7	0.379807692	0.016695124
EPHX1	7	0.25566343	0.009271881
MAPK14	7	0.355855856	0.003075791
GSTP1	6	0.32780083	0.20453316
CYP1B1	6	0.25483871	0.009217786
GSTM1	6	0.207894737	5.41E-05

GSTM2	6	0.207894737	5.41E-05
MMP2	5	0.370892019	0.07271022
CDK2	5	0.30859375	0.014047408
MALT1	5	0.321138211	0.006633247
MAP2K1	5	0.344978166	0.001443801
ESR2	5	0.351111111	9.56E-04
FLT3	5	0.369158879	6.98E-04
PIK3CG	5	0.333333333	0
IL1B	4	0.370892019	0.027683597
CDK4	4	0.30859375	0.013194627
DNMT1	4	0.36744186	0.011181826
LCK	4	0.369158879	0.009724465
COL18A1	3	0.311023622	0.004338164
ICAM1	3	0.333333333	0.002101275
PARP1	3	0.314741036	0.001796726
BRD4	3	0.301526718	0
CHRNA4	3	0.293680297	0
CHRNB4	3	0.293680297	0
CHRNA3	3	0.293680297	0
ALDH1A1	3	0.206266319	0
IKBKB	2	0.312252964	0.001267585
TERT	2	0.318548387	1.11E-04
ABL1	2	0.318548387	1.11E-04
PTPRC	2	0.279151943	1.08E-04
PTGS2	2	0.355855856	0
IGFBP3	2	0.311023622	0
EPAS1	2	0.299242424	0
NFE2L2	2	0.299242424	0
DNMT3A	2	0.291512915	0
MMP1	2	0.278169014	0
CCNE1	2	0.245341615	0
MPO	2	0.233038348	0
ELANE	2	0.233038348	0
CYP2D6	2	0.205729167	0
KDM5C	1	0.290441176	0
KDM5A	1	0.290441176	0
MMP7	1	0.271477663	0
MCL1	1	0.269624573	0

Table S8: This table highlights 11 proteins identified through network analysis and their interconnection to 14 enriched lung cancer pathways. The proteins present in the pathways are represented using tick marks, while blanks indicate no interaction with pathways. Among these, EGFR stands out as a key player, interacting with 12 pathways more than any other protein. This suggests that inhibiting EGFR could potentially have a significant impact on reducing the activity of pathways linked to lung cancer.

	**Pathways in cancer**	**FoxO signaling pathway**	**JAK-STAT signaling pathway**	**HIF-1 signaling pathway**	**PD-L1 expression and PD-1 checkpoint pathway in cancer**	**Th17 cell differentiation**	**Wnt signaling pathway**	**PI3K-Akt signaling pathway**	**Non-small cell lung cancer**	**Rap1 signaling pathway**	**ErbB signaling pathway**	**Ras signaling pathway**	**GnRH signaling pathway**	**MAPK signaling pathway**
**EGFR**	✓	✓	✓	✓	✓			✓	✓	✓	✓	✓	✓	✓
**STAT3**	✓	✓	✓	✓	✓	✓			✓					
**ESR1**	✓													
**MAPK8**	✓	✓				✓	✓				✓	✓	✓	✓
**IGF1R**	✓	✓		✓				✓		✓		✓		✓
**CTNNB1**	✓						✓			✓				
**CREBBP**	✓	✓	✓	✓			✓							
**HSP90AA1**	✓					✓		✓						
**HRAS**	✓	✓	✓		✓			✓	✓	✓	✓	✓		✓
**JAK2**	✓		✓		✓	✓		✓					✓	
**EP300**	✓	✓	✓	✓			✓							

Table S9: This table highlights 11 proteins identified through network analysis and their interaction to 10 compounds. Notably, EGFR is targeted by most of the selected M. oleifera bioactive compounds.

	**EGFR**	**STAT3**	**ESR1**	**MAPK8**	**IGF1R**	**CTNNB1**	**CREBBP**	**HSP90AA1**	**HRAS**	**JAK2**	**EP300**
Vanillin											✓
p-Coumaric acid	✓	✓	✓								
Caffeic Acid	✓	✓	✓								✓
cis-3-Hexen-1-ol										✓	
trans-Linalool oxide					✓					✓	
cis-3-Hexenyl acetate	✓									✓	
cis-Linalool Oxide					✓					✓	
Moringyne	✓							✓	✓		
Gibberellic acid				✓			✓				
Gibberellin A29	✓			✓	✓	✓	✓				

Table S10: This table contains docking information of 10 compounds with proteins identified through network analysis and their (-) CDOCKER Energy in kcal/mol. The green colored cells inticate that the corresponding protein is already experimentally validated as caffeic acid target protein in previous studies [51–57].

	EGFR	HRAS	STAT3	IGFR1	MAPK8	JACK2	CREBBP	EP300	HSP90AA1	CTNNB1	ESR1
Caffeic Acid	28.972[52]	31.544	23.6476[51]	30.0324[55]	29.806[57]	30.1238[56]	28.1085	33.943	29.8711[54]	25.0514[53]	29.8802
p-Coumaric acid	26.2252	27.2378	20.4906	23.9206	25.5208	21.4546	27.6654	27.4278	24.6221	21.9698	24.5693
Vanillin	21.9652	25.1003	15.1947	21.8741	24.2202	22.6686	23.454	24.5366	23.0881	19.3383	22.8676
cis-3-Hexenyl acetate	14.4486	13.6702	11.3139	15.6069	11.942	10.9415	10.0295	15.0698	11.2601	10.0329	11.297
Moringyne	13.0136	12.3839	-9.22677	9.89321	18.8606	12.4156	5.09414	10.4804	9.77604	6.51332	3.70696
cis-Linalool Oxide	2.84052	6.17098	-8.57937	2.82492	-1.80903	-0.436433	1.09733	4.38275	1.55514	2.69613	2.69564
cis-3-Hexen-1-ol	2.24304	3.80325	2.53597	2.96659	4.62359	3.76205	5.1834	5.10586	4.52341	2.69613	6.12363
trans-Linalool oxide	1.1452	5.16515	-1.73447	2.57032	1.06566	3.96103	-0.84066	4.49367	0.27762	1.13323	3.53698
Gibberellin A29	-46.364	-32.7553	0	-34.8435	-40.5358	-46.5187	-52.1051	-58.0037	-41.0521	-45.6483	-45.9503
Gibberellic acid	-65.322	-57.1651	0	-60.6529	-58.7453	-69.9288	-71.7548	-71.7796	-60.5153	-64.3162	-64.2109

51. Jung JE, Kim HS, Lee CS, Park DH, Kim YN, Lee MJ, Lee JW, Park JW, Kim MS, Ye SK, Chung MH. Caffeic acid and its synthetic derivative CADPE suppress tumor angiogenesis by blocking STAT3-mediated VEGF expression in human renal carcinoma cells. Carcinogenesis. 2007 Aug;28(8):1780-7. doi: 10.1093/carcin/bgm130. Epub 2007 Jun 8.

52.. Yuan JW, Qiu HY, Wang PF, Makawana JA, Yang YA, Zhang F, Yin Y, Lin J, Wang ZC, Zhu HL. Synthesis of caffeic acid amides bearing 2,3,4,5-tetra-hydrobenzo[b][1,4]dioxocine moieties and their biological evaluation as antitumor agents. Molecules. 2014 Jun 3;19(6):7269-86. doi: 10.3390/molecules19067269.;.

53. Villota H, Santa-González GA, Uribe D, Henao IC, Arroyave-Ospina JC, Barrera-Causil CJ, Pedroza-Díaz J. Modulatory Effect of Chlorogenic Acid and Coffee Extracts on Wnt/β-Catenin Pathway in Colorectal Cancer Cells. Nutrients. 2022 Nov 18;14(22):4880. doi: 10.3390/nu14224880. PMCID: PMC9693551.

54. Alam M, Abbas K, Ahmad A, Showkat N, Sen R. Identification of Hsp90 inhibitors from Ananas comosus potential phytochemicals for lung cancer treatment. J Phytopharmacol 2024; 13(1):12-19. doi: 10.31254/phyto.2024.13103

55. Hou L, Ma J, Feng X, Chen J, Dong BH, Xiao L, Zhang X, Guo B. Caffeic acid and diabetic neuropathy: Investigating protective effects and insulin-like growth factor 1 (IGF-1)-related antioxidative and anti-inflammatory mechanisms in mice. Heliyon. 2024 Jun 6;10(12):e32623. doi: 10.1016/j.heliyon.2024.e32623.; PMCID: PMC11225750.

56. Li PG, Xu JW, Ikeda K, Kobayakawa A, Kayano Y, Mitani T, Ikami T, Yamori Y. Caffeic acid inhibits vascular smooth muscle cell proliferation induced by angiotensin II in stroke-prone spontaneously hypertensive rats. Hypertens Res. 2005 Apr;28(4):369-77. doi: 10.1291/hypres.28.369. PMID: 16138568.

57. Kang NJ, Lee KW, Shin BJ, Jung SK, Hwang MK, Bode AM, Heo YS, Lee HJ, Dong Z. Caffeic acid, a phenolic phytochemical in coffee, directly inhibits Fyn kinase activity and UVB-induced COX-2 expression. Carcinogenesis. 2009 Feb;30(2):321-30. doi: 10.1093/carcin/bgn282. Epub 2008 Dec 10. PMID: 19073879;.

Table S11: The bonds information after the molecular docking of caffeic acid & erlotinib with EGFR.

**EGFR_Caffeic acid (Molecular Docking)**			
**Bond Name**	**Distance**	**Category**	**Type**
Caffeic acid:O2- EGFR:LYS721:HZ2	1.91234	Hydrogen Bond	Conventional Hydrogen Bond
Caffeic acid:O3- EGFR:MET769:HN	2.36595	Hydrogen Bond	Conventional Hydrogen Bond
Caffeic acid:H14 - EGFR:GLU738:OE2	2.8213	Hydrogen Bond	Conventional Hydrogen Bond
Caffeic acid:H15 - EFGR:GLU738:OE2	2.15002	Hydrogen Bond	Conventional Hydrogen Bond
Caffeic acid:H16 - EGFR:GLN767:O	2.574	Hydrogen Bond	Conventional Hydrogen Bond
Caffeic acid:O3 - EGFR:LEU768:HA	2.47142	Hydrogen Bond	Carbon Hydrogen Bond
Caffeic acid:O1 - EGFR:MET742:SD	3.19663	Other	Sulfur-X
Caffeic acid - EGFR:THR830:HG1	3.06624	Hydrogen Bond	Pi-Donor Hydrogen Bond
Caffeic acid - EGFR:MET742:SD	5.80614	Other	Pi-Sulfur
Caffeic acid - EGFR:LYS721	5.00645	Hydrophobic	Pi-Alkyl
**EGFR_Erlotinib (Molecular Docking)**			
**Bond Name**	**Distance**	**Category**	**Types**
Erlotinib:O17- EGFR:THR766:HG1	2.50699	Hydrogen Bond	Conventional Hydrogen Bond
Erlotinib:O14 - EGFR:ASP831:HA	2.30609	Hydrogen Bond	Carbon Hydrogen Bond
Erlotinib:H34 - EGFR:ASP831:OD2	2.40412	Hydrogen Bond	Carbon Hydrogen Bond

Erlotinib:H38 - EGFR:GLU738:OE2	2.56887	Hydrogen Bond	Carbon Hydrogen Bond
Erlotinib:H39 - EGFR:THR830:OG1	2.46937	Hydrogen Bond	Carbon Hydrogen Bond
Erlotinib:H43 - EGFR:LEU764:O	2.65346	Hydrogen Bond	Carbon Hydrogen Bond
Erlotinib:H49 - EGFR:MET769:O	2.54807	Hydrogen Bond	Carbon Hydrogen Bond
Erlotinib - EGFR:ALA719	4.93315	Hydrophobic	Pi-Alkyl
Erlotinib - EGFR:LEU820	4.12694	Hydrophobic	Pi-Alkyl
Erlotinib - EGFR:VAL702	4.68842	Hydrophobic	Pi-Alkyl
Erlotinib - EGFR:ALA719	4.51736	Hydrophobic	Pi-Alkyl
Erlotinib- EGFR:LEU820	4.80445	Hydrophobic	Pi-Alkyl

Table S12: The bonds information after MD simulation of EGFR with caffeic acid and erlotinib.

EGFR_Caffeic Acid (MD simulation)			
**Bond Name**	**Distance**	**Category**	**Types**
Caffeic acid:O4 - EGFR:MET769:HN	2.70689	Hydrogen Bond	Conventional Hydrogen Bond
Caffeic acid: H16 - EGFR: THR766:O	2.1611	Hydrogen Bond	Conventional Hydrogen Bond
Caffeic acid:O3 - EGFR:LEU768:HA	2.8358	Hydrogen Bond	Carbon Hydrogen Bond
Caffeic acid:O2- EGFR:ASP831:HA	2.2477	Hydrogen Bond	Carbon Hydrogen Bond
Caffeic acid - EGFR:LYS721:HD2	2.63823	Hydrophobic	Pi-Sigma
**EGFR_Erlotinib (MD simulation)**			
**Bond Name**	**Distance**	**Category**	**Types**
Erlotinib: O14- EGFR:LYS721:HZ1	1.98889	Hydrogen Bond	Conventional Hydrogen Bond
Erlotinib: O14- EGFR:LYS721:HZ2	2.41721	Hydrogen Bond	Conventional Hydrogen Bond
Erlotinib: N26- EGFR:LYS704:HE1	2.50357	Hydrogen Bond	Carbon Hydrogen Bond
Erlotinib: N26-EGFR:LYS704:HE2	2.79346	Hydrogen Bond	Carbon Hydrogen Bond
Erlotinib: H49 - EGFR: GLY772:O	2.44051	Hydrogen Bond	Carbon Hydrogen Bond
Erlotinib:C1- EGFR:CYS773	3.25926	Hydrophobic	Alkyl
Erlotinib- EGFR:LEU694	4.72946	Hydrophobic	Pi-Alkyl
Erlotinib - EGFR:VAL702	4.87734	Hydrophobic	Pi-Alkyl
Erlotinib - EGFR:CYS773	4.17882	Hydrophobic	Pi-Alkyl
Erlotinib - EGFR:ALA719	3.4633	Hydrophobic	Pi-Alkyl
Erlotinib - EGFR:LEU820	5.25304	Hydrophobic	Pi-Alkyl
Erlotinib - EGFR:ALA719	3.27267	Hydrophobic	Pi-Alkyl

Table S13: Table illustrates 10 bioactive compounds after the ADMET analysis and their concentration in M. oleifera.

Compound	Concentration	Reference
Caffeic acid	2.43 mg/g	Oldoni et al,2022[76]
p-coumaric acid	1.25mg/g	Mumtaz et al,2021[20]
Vanillin	0.02619mg/g	Zhu et al., 2020[77]
Gibberellic acid	0.001 mg/g	Cirlini et al., 2022[80]
cis-3-Hexenyl acetate	No data available	-
Moringyne	0.714 mg/g	Memon et al,1985[78]
cis-Linalool Oxide	In traces	Chuang et al., 2007[79]
cis-3-Hexen-1-ol	No data available	-
trans-Linalool oxide	In traces	Chuang et al., 2007[79]
Gibberellin A29	No data available	-

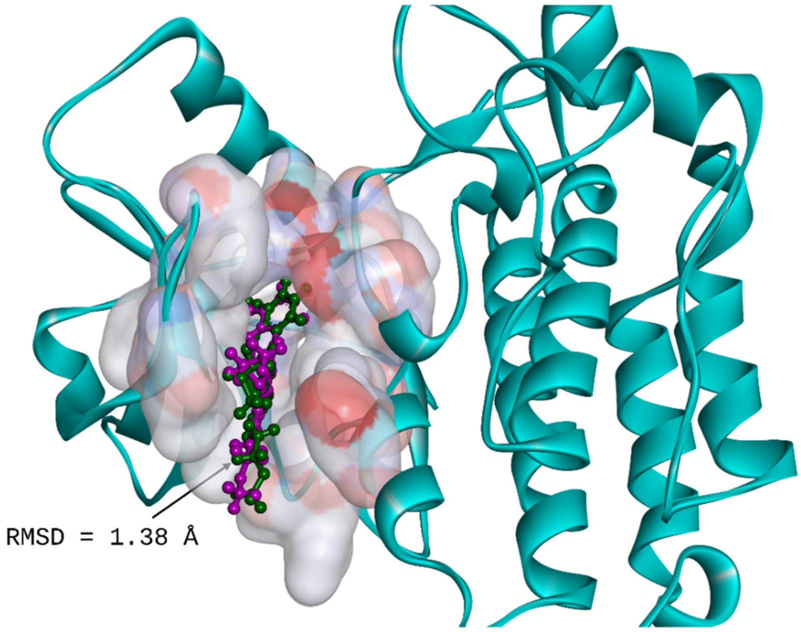

Figure S1: Superimposed view of erlotinib in its crystallographic and redocked conformation. Erlotinib from the crystallographic structure is shown in green, while the redocked conformation is depicted in purple. The protein EGFR is represented in cyan, and the surface of the active site is coloured based on atom charges.

.
